# Neuromodulation of the Feedforward Dentate Gyrus-CA3 Microcircuit

**DOI:** 10.3389/fnsyn.2016.00032

**Published:** 2016-10-17

**Authors:** Luke Y. Prince, Travis J. Bacon, Cezar M. Tigaret, Jack R. Mellor

**Affiliations:** Centre for Synaptic Plasticity, School of Physiology, Pharmacology and Neuroscience, University of BristolBristol, UK

**Keywords:** acetylcholine, noradrenaline, dopamine, serotonin, mossy fiber, dentate gyrus, CA3, computational modeling

## Abstract

The feedforward dentate gyrus-CA3 microcircuit in the hippocampus is thought to activate ensembles of CA3 pyramidal cells and interneurons to encode and retrieve episodic memories. The creation of these CA3 ensembles depends on neuromodulatory input and synaptic plasticity within this microcircuit. Here we review the mechanisms by which the neuromodulators aceylcholine, noradrenaline, dopamine, and serotonin reconfigure this microcircuit and thereby infer the net effect of these modulators on the processes of episodic memory encoding and retrieval.

## 1. Introduction

The hippocampus has been strongly implicated in encoding and retrieval of memories since the early work of Scoville and Milner several decades ago (Scoville and Milner, 1957; Squire, 1992), but the mechanisms through which this is achieved remain largely elusive. The hippocampal circuit receives input from the entorhinal cortex and transforms this information through successive synaptic transmission in feedforward and recurrent networks before projecting back to the entorhinal cortex. Theoretical investigations have shown that this transformation through a succession of feedforward and recurrent networks is well suited to the encoding and retrieval of memories in neural structures (Marr, 1971; McNaughton and Morris, 1987; Treves and Rolls, 1994; Lengyel et al., 2005).

**Figure 1:**
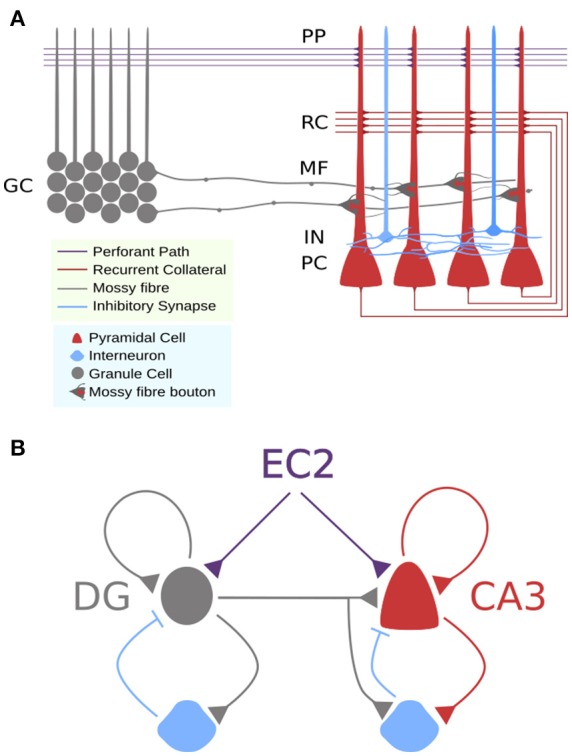
**Circuit schemas for DG-CA3 microcicuit. (A)** Detailed schematic showing approximate locations of synaptic connections to CA3 pyramidal cells and differences in axon terminals. **(B)** Simplified schematic showing feedforward and recurrent circuits with feedforward and feedback inhibition.

The dentate gyrus and CA3 subfields are central to these processes. Dentate gyrus granule cells project to recurrently connected CA3 pyramidal cells and this microcircuit (Figure 1) has been shown to contribute to learning contexts (McHugh et al., 2007; Kheirbek et al., 2013) and detecting novelty (Hunsaker et al., 2007, 2008), which are key requirements for episodic and recognition memory. However, encoding and retrieval engage distinct processes as demonstrated by evidence that contralateral lesions to dentate gyrus and CA3 impaired encoding, but not retrieval in a spatial maze learning task relative to ipsilateral lesioned and unlesioned animals (Jerman et al., 2006) whilst damage to direct entorhinal-CA3 inputs impairs retrieval but not encoding (Lee and Kesner, 2004). These and other data support a long held hypothesis of a double dissociation in encoding and retrieval pathways to CA3 (Treves and Rolls, 1994).

Episodic memories are thought to be encoded in CA3 by the distribution of synaptic efficacies derived through activation of long-term synaptic plasticity mechanisms by correlated spiking (Hebb, 1949; Mishra et al., 2016). These distributions of synaptic weights form sparse orthogonalized cell assemblies that are reactivated during memory retrieval with high precision even with partial or corrupted cues (O'Reilly and McClelland, 1994), and retain sufficient flexibility to allow memory sequences to be retrieved when required (Lisman, 1999). Together, encoding and retrieval is supported by the processes of pattern separation and completion, where separation refers to a decrease in output correlation relative to the input and supports optimal encoding, and completion refers to an increase in output correlation relative to the input and supports precise retrieval (O'Reilly and McClelland, 1994; Fuhs and Touretzky, 2000; Nolan et al., 2011). Although theories have often posited that the dentate gyrus performs pattern separation and CA3 performs pattern completion (O'Reilly and McClelland, 1994), more recent experimental evidence suggests that both subfields can perform both functions (Leutgeb et al., 2007).

An important and often ignored component of memory encoding and retrieval is the neuromodulatory influence of acetylcholine (ACh), dopamine (DA), noradrenaline (NA), and serotonin (5-HT). Each of these neuromodulators are released in the hippocampus and have been suggested to encode information pertaining to reward, novelty, salience, and surprise, all of which are relevant for optimizing processes involved in encoding and retrieval of information when traversing an uncertain environment with limited computational resources. To date there have been few theoretical efforts to integrate the actions of neuromodulators in memory encoding and retrieval. In this article, we propose potential mechanisms through which encoding and retrieval can be modulated by considering observed and putative effects on cellular and synaptic properties throughout the DG-CA3 microcircuit.

## 2. Circuit mechanisms for memory encoding and retrieval in the CA3 region of the hippocampus

### 2.1. Dentate gyrus output and function

The dentate gyrus (DG) consists of a large population (1,000,000 in rats; West et al., 1991) of tightly packed granule cells and interneurons. Computational theories of hippocampal function have argued that the connectivity and dynamics of this structure lend themselves to efficient decorrelation of input from layer II of entorhinal cortex, whereby strong lateral inhibition ensures very few granule cells are allowed to fire at any given time, limiting the possibility of interference between stimuli or events with similar features by accentuating small differences (O'Reilly and McClelland, 1994; Leutgeb et al., 2007). Granule cells are often observed to have low average firing rates, and bimodal firing rate distributions with long periods of quiescence punctured by very high frequency bursts (Jung and McNaughton, 1993; Mistry et al., 2011). However, more recent studies provide evidence for subpopulations of granule cells that fire with more unimodal low frequency firing rates (Mistry et al., 2011), with electrophysiological and functional differences between populations found to correlate with maturity (Marín-Burgin et al., 2012; Nakashiba et al., 2012; Danielson et al., 2016). Adult born granule cells appear more excitable and less input specific yet better suited to pattern separation, whilst mature granule cells were found to be less excitable and more input specific yet better suited to pattern completion supporting a role for the dentate gyrus in both processes via separate populations of granule cells.

### 2.2. Granule cell inputs to CA3 pyramidal cells

Granule cell axons project sparsely to CA3 pyramidal cells (1 granule cell onto around 37 out of 300,000 pyramidal cells in rats; West et al., 1991; Acsády et al., 1998) and more diffusely to CA3 interneurons in the stratum lucidum. These axons, referred to as mossy fibers due to prominent en passant and large boutons, terminate onto CA3 pyramidal cells with large thorny excrescences located on the proximal region of apical and basal dendrites (Acsády et al., 1998; Wilke et al., 2013). These synapses possess 18-45 release sites and have enormous capacity for vesicle storage (Wilke et al., 2013). They are also noted for having low initial release probability but a wide time window for presynaptic facilitation of a few milliseconds to several seconds (Salin et al., 1996; Toth et al., 2000; Gundlfinger et al., 2007). Sufficiently long, albeit realistic, bursts of granule cell firing can elicit a presynaptic form of LTP that raises basal release probability but reduces the range of presynaptic short-term facilitation (Zalutsky and Nicoll, 1990; Salin et al., 1996; Gundlfinger et al., 2007, 2010; Mistry et al., 2011). Collectively, this means the synapse has huge capacity for gain control over a wide range of input frequencies and has often been referred to as a “conditional detonator” due to its ability to elicit spikes in response to activity at a single synapse contingent on repeated stimulation.

### 2.3. Granule cell inputs to a feedforward interneuron population

Mossy fibers excite a diverse population of inhibitory interneurons located in the stratum lucidum, forming synapses via en passant boutons and small filopodia which protrude from large boutons directly onto interneuron dendrites (Acsády et al., 1998; Wilke et al., 2013), and this interneuron population has been shown to contribute to precision of memory encoding and retrieval (Ruediger et al., 2011; Restivo et al., 2015). Mossy fibers excite 10 times more interneurons than pyramidal cells, thus exerting strong widespread feedforward inhibitory control via a disynaptic pathway that prevents runaway excitation and ensures tight spike timing (Acsády et al., 1998; Mori et al., 2007; Torborg et al., 2010; Treviño et al., 2011).

However the identity and contribution of interneuron subtypes to feedforward mossy fiber driven inhibition is somewhat unclear. Szabadics and Soltesz (2009) identified ivy cells, parvalbumin-positive (PV+) fast spiking basket cells, and cholecystokinin-positive (CCK+) regular spiking basket cells as key contributing interneurons, while Toth et al. (2000) found a separation between interneurons with calcium permeable (CP) or impermeable (CI) AMPA receptors. The overlap between these subtypes in CA3 is unclear, but in other hippocampal regions CP-AMPARs are preferentially expressed on PV+ interneurons, and CI-AMPARS on CCK+ interneurons (Nissen et al., 2010). Characteristics of these cell types are summarized in Table 1.

**Table 1:** **Characteristics of mossy fiber feedforward interneuron-pyramidal cell plasticity in CA3**.

**Interneuron subtype**	**Forms of interneuron-pyramidal cell plasticity**
CI-AMPAR containing	Short-term facilitation of MF input (Toth et al., 2000). NMDAR-dependent LTD in response to high frequency stimulation via NMDARs (Maccaferri et al., 1998).
CP-AMPAR containing	Voltage dependent short term facilitation of MF input (Toth et al., 2000). mGluR7 -dependent LTD in response to high frequency stimulation (Pelkey et al., 2005).
PV+ FSBC	Weak short-term facilitation of mossy fiber input (Szabadics and Soltesz, 2009). Short-term depressing synchronous GABA release (Szabó et al., 2010).
CCK+ RSBC	Strongly short-term depressing mossy fiber input (Szabadics and Soltesz, 2009). Short-term facilitating GABA release (Szabó et al., 2010). Asynchronous GABA release at high firing frequencies (Szabó et al., 2010).
Ivy	Strong short-term depressing mossy fiber input. Dendritic targeting, dense axonal arborization (Szabadics and Soltesz, 2009).

These interneuron subtypes may perform distinct functional roles. Although PV+ basket cells receive weak mossy fiber input, they also receive a high rate of spontaneous excitation that may tonically depolarize them such that weak fluctuations are sufficient to synchronously activate a subset of their total population to provide tight control of pyramidal cell spike timing (Szabadics and Soltesz, 2009; Szabó et al., 2010; Torborg et al., 2010). However, mossy fiber drive to the PV+ basket cell network in CA3 can be vastly strengthened during learning by increased filopodial growth from mossy fiber boutons onto these interneurons (Ruediger et al., 2011; Donato et al., 2013). On the other hand, CCK+ interneurons are excited by much stronger mossy fiber input but release GABA asynchronously, providing a tonic shunt, which may be reduced by release of endocannabinoids from CA3 pyramidal cells caused by an increase in spike frequency (Losonczy et al., 2004; Szabadics and Soltesz, 2009; Szabó et al., 2010). Finally, ivy cells target more distal portions of dendrites compared to basket cells and mossy fibers, which may provide some compartmental isolation in favor of mossy fiber transmission by suppressing the influence of upstream dendritic activation by competing excitatory pathways. These putative functional roles for interneuron subtypes are still largely speculative and are key areas for investigation.

### 2.4. The CA3 pyramidal cell network

Feedforward monosynaptic excitation and disynaptic inhibition by mossy fiber input combine to selectively activate a small subpopulation of CA3 pyramidal cells (Mori et al., 2004) driving a transient phase coupling of DG-CA3 subnetworks (Akam et al., 2012). Many researchers have proposed that recurrent synapses amongst CA3 pyramidal cells endow the CA3 network with autoassociative and heteroassociative properties (Marr, 1971; Treves and Rolls, 1994; Lisman, 1999; Bush et al., 2010; Savin et al., 2014; Guzman et al., 2016) such that ensembles of strongly associated CA3 pyramidal cells can be reactivated when only a subset of their constituent CA3 pyramidal cells have been activated by external sources. This mechanism is used to store and retrieve memories (Treves and Rolls, 1994; Lisman, 1999; Bush et al., 2010) via NMDA receptor (NMDAR)-dependent Hebbian plasticity (Nakazawa et al., 2004; Nakashiba, 2008; Mishra et al., 2016) that encodes memories as configurations of synaptic efficacies in the network, and retrieves them by precisely timed reactivation of cell ensembles stored as network motifs within these configurations. Stability, precision, and efficiency of this mechanism amidst uncertainty is supported by local oscillations that isolate subnetworks and encode memories by phase, and through subtle changes to intrinsic excitability of neurons during encoding and retrieval (Kunec et al., 2005; Lengyel et al., 2005; Lengyel and Dayan, 2007; Savin et al., 2014). Since mossy fiber synapses provide highly targeted sparse activation of subpopulations of CA3 pyramidal cells they are believed by some to be capable of driving both encoding and retrieval, by imposing synchronization necessary for driving Hebbian plasticity between CA3 pyramidal cells to establish ensembles (Kobayashi and Poo, 2004; Brandalise and Gerber, 2014; Mishra et al., 2016). Mossy fibers can also reactivate these CA3 ensembles at a later timepoint by triggering a local cascade of transient ensemble synchronization.

## 3. Neuromodulation of DG-CA3 microcircuit

### 3.1. Acetylcholine

#### 3.1.1. Release and receptors

Acetylcholine affects the dentate gyrus-CA3 microcircuit through the activity of muscarinic and nicotinic ACh receptors, and is delivered to the hippocampus by cholinergic cells projecting from the medial septum. Cholinergic fibers innervate the stratum oriens of CA3 and inner molecular layer of the dentate gyrus, and thin fibers have been found to innervate the stratum lucidum and hilar region (Kitt et al., 1994; Grybko et al., 2011).

Four muscarinic receptor subtypes are expressed in the hippocampus, but the main effects are driven through M1 and M2 receptors. M1 receptors are located on somatic and dendritic membranes of dentate gyrus granule cells and CA3 pyramidal cells (Levey et al., 1995; Dasari and Gulledge, 2011; Martinello et al., 2015), on mossy fibers (Martinello et al., 2015), and PV+ basket cells (Chiang et al., 2010; Yi et al., 2014). These receptors couple to Gq proteins and stimulate the phospholipase C signaling pathway to increase intracellular IP3 and DAG. M2 receptor expression has been found on stratum lucidum interneurons, and PV+ basket cell axon terminals (Levey et al., 1995; Hájos et al., 1998) and sparsely on CA3 pyramidal cells (Levey et al., 1995), coupling to Gi proteins that reduce intracellular cAMP.

Nicotinic receptors are ligand gated cation channels with different functions depending on their affinity for ACh, desensitization kinetics, and permeability to calcium ions. Nicotinic receptors composed of five α7 subunits are widespread in the hippocampus, and have a high permeability to calcium ions, low affinity for nicotine, and undergo rapid desensitization. α7 receptors have been found on DG and CA3 interneurons (Son and Winzer-Serhan, 2008), on granule cell somas and dendrites, and sparsely in the stratum radiatum and stratum lucidum (Fabian-Fine et al., 2001). Furthermore, functional nicotinic receptors are more likely to be expressed on immature granule cells, and interneurons close to them (John et al., 2015). Although expression appears to be sparse, functional α7 nicotinic receptors have been detected in CA3 pyramidal cells, stratum lucidum interneurons, and mossy fiber synapses (Radcliffe et al., 1999; Frazier et al., 2003; Sharma and Vijayaraghavan, 2003; Sharma et al., 2008; Grybko et al., 2010, 2011). The evidence for other nicotinic receptor subtypes is less strong, although functional α4β2 and α3β4 receptors have been found on interneurons in CA1 (Jones and Yakel, 1997; McQuiston and Madison, 1999; Alkondon and Albuquerque, 2001; but see Gahring et al., 2004; Gahring and Rogers, 2008).

#### 3.1.2. Muscarinic effects on cellular and synaptic properties

Excitability of granule cells, CA3 pyramidal cells, and PV+ basket cells, is strongly modulated by muscarinic activity. M1 receptors increase excitability and lower spike threshold of these cells by suppressing sAHP currents (Vogt and Regehr, 2001; Chiang et al., 2010; Dasari and Gulledge, 2011) and Kv7 (Martinello et al., 2015), thereby increasing the gain on any excitatory input. Furthermore, in CA3 pyramidal cells M1 receptors contribute to robust persistent firing following brief depolarization by activation of calcium sensitive non-selective ion channels (Jochems and Yoshida, 2013, 2015).

Muscarinic receptors also affect synaptic transmission in this circuit. M1 receptors indirectly suppress recurrent glutamatergic transmission between CA3 pyramidal cells, and GABAergic transmission from CCK+ basket cells by stimulating the release of endocannabinoids from pyramidal cells, mossy cells, and granule cells to reduce presynaptic calcium influx and suppress vesicle release via presynaptic CB1 receptors (Hasselmo et al., 1995; Scanziani et al., 1995; Vogt and Regehr, 2001; Fukudome et al., 2004; Hofmann et al., 2006, 2008; Hofmann and Frazier, 2010; Szabó et al., 2010). Furthermore, M2 receptors suppress release from PV+ basket cells in DG and CA3 (Chiang et al., 2010; Szabó et al., 2010). These effects of muscarinic receptors collectively dampen recurrent activity in CA3, leaving cell dynamics to be driven primarily by feedforward and intrinsic mechanisms (Hasselmo et al., 1995; Jochems and Yoshida, 2015).

The mossy fiber synapse is indirectly modulated by muscarinic receptor activity. Due to increased granule cell firing frequency mossy fiber synapses become stronger through frequency facilitation (Vogt and Regehr, 2001). On the other hand, due to increased interneuron firing frequency, build up of extrasynaptic GABA concentration weakly suppresses mossy fiber synapses through GABA-B receptor activation (Vogt and Regehr, 2001; Chandler et al., 2003; Nahir et al., 2007). Additionally, since extrasynaptic GABA can also help induction of mossy fiber LTP and enhance transmission through modulation of calcium influx by high affinity delta subunit containing GABA-A receptors (Ruiz et al., 2010), the effect of extrasynaptic GABA may be biphasic. Since these processes occur on different time scales, it may be expected to see a transient indirect potentiation of mossy fiber synapses, followed by suppression in response to tonic muscarinic activity. These indirect effects on mossy fiber plasticity can account for experiments suggesting a direct effect of muscarinic receptors (Williams and Johnston, 1988, 1990).

M1 receptor activity has also been demonstrated to be key to enabling Hebbian NMDAR-dependent LTP between pyramidal cells in the hippocampus by inhibition of SK channels to increase spine calcium influx (Müller and Connor, 1991; Buchanan et al., 2010; Giessel and Sabatini, 2010). This effect on synaptic plasticity has mainly been studied in CA1, and some differences with CA3 have been found. For example, CA3 pyramidal cells internalize more NMDARs in response to increases in intracellular calcium concentration through IP3 activation, which can induce LTD (Grishin et al., 2004, 2005). Additionally, muscarinic receptors have been implicated in inhibitory depression onto pyramidal cells through coactivation of presynaptic M2 and CB1 receptors that reduce GABA release by activation of cAMP/PKA signaling pathways (Ahumada et al., 2013). Notably, this form of plasticity was induced by the same theta-burst stimulation protocol that induces potentiation of glutamatergic synapses. However, this is counterbalanced by a postsynaptic inhibitory potentiation enabled by M1 receptors that causes an enhancement of α5βγ2 GABA-A receptors influencing sensitivity to both CCK+ and PV+ basket cells (Domínguez et al., 2014, 2015).

#### 3.1.3. Nicotinic effects on cellular and synaptic properties

The functional role of nicotinic receptors in this circuit is less clear cut. In CA3, α7 nicotinic receptors have small effects on recurrent excitatory and inhibitory synapses onto pyramidal cells, which complement muscarinic effects (Giocomo and Hasselmo, 2005; Liotta et al., 2011; Fischer et al., 2014). In the dentate gyrus, nicotine enhances LTP at medial perforant path (mPP)-DG synapses through α7 receptors, and co-activation of NMDA and mGluR5 to activate the ERK-PKA signaling pathway (Welsby et al., 2006, 2009; Ondrejcak et al., 2012). This LTP is likely to be suppressed by local inhibition that is further strengthened by nicotinic receptors (Frazier et al., 2003), but interestingly this may be opposed by recruitment of mossy cells to provide a selective local excitatory boost that may be sufficient to enable LTP amongst a smaller number of synapses (Hofmann and Frazier, 2010).

A striking effect of nicotinic receptors within this circuit is stimulation of action potential independent synaptic release. At mossy fiber synapses, despite low expression seen in stratum lucidum (Fabian-Fine et al., 2001), α7 receptor activity induces bursts of action potential independent multiquantal release by stimulation of calcium induced calcium release that is sufficient to elicit postsynaptic action potentials (Sharma and Vijayaraghavan, 2003; Sharma et al., 2008). Additionally, over a longer time scale, activity of α7 receptors have been shown to enhance mossy fiber release via PKA activation (Cheng and Yakel, 2014). Action potential independent release stimulated by nicotinic receptors has also been shown in interneurons, albeit through a different mechanism. Tang et al. (2011) demonstrated that α3β4 receptors induce GABA release at PV+ basket cell synapses by activating T-type calcium channels and calcium induced calcium release.

Nicotinic receptors may have a further role at mossy fiber synapses. Structural plasticity of mossy fiber synapses through growth of filopodia onto PV+ interneurons occurs during contextual fear conditioning tasks (Ruediger et al., 2011), and nicotinic receptors have been implicated in filopodial growth at thalamic axon growth cones (Rüdiger and Bolz, 2008). Since the dentate gyrus continually integrates immature granule cells into its population, these cells may possess machinery for nicotinic mediated filopodial growth, which may be supported by greater α7 expression in immature granule cells (John et al., 2015).

#### 3.1.4. Summary

- Muscarinic receptors combine to directly increase cellular excitability in granule cells, CA3 pyramidal cells, and interneurons.
- Muscarinic receptors depress synaptic transmission at inhibitory synapses and recurrent excitatory synapses.
- Muscarinic receptors lower requirements for Hebbian LTP induction.
- Muscarinic receptors indirectly enhance and suppress mossy fiber synapses over different time scales.
- Nicotinic receptors enable (mPP)-DG LTP.
- Nicotinic receptors can induce action potential independent release at mossy fiber synapses.

### 3.2. Noradrenaline

#### 3.2.1. Noradrenaline release and receptors

Noradrenaline is released into the hippocampus from locus coeruleus (LC) projections, with strong direct innervation onto granule cells and hilar mossy cells in the dentate gyrus, CA3 pyramidal cells, and PV+ interneurons in the stratum lucidum (Hörtnagl et al., 1991; Milner et al., 2000; Walling et al., 2012). LC firing is tightly correlated with behavioral state, and novel or emotive stimuli induce burst firing of LC neurons (Vankov et al., 1995), with the nature and saliency of the experience influencing the intensity and duration of firing (Sara, 2009).

NA influences hippocampal function through activation of three classes of adrenoreceptors: α1-, α2-, and β-ARs, all of which are G-protein coupled: α1-ARs are coupled to Gq proteins and increase intracellular IP3 and DAG; α2-ARs couple to Gi proteins that inhibit adenylyl cyclase and reduce intracellular cAMP; whereas β-ARs stimulate Gs proteins that subsequently activate adenylyl cyclase and increase intracellular cAMP. β-ARs in CA3 show expression on cell membranes at somas and proximal dendrites of pyramidal cells, and subcellularly in the cytoplasm and nucleus (Jurgens et al., 2005; Guo and Li, 2007). Pre- and postsynaptic expression has been reported in the dentate gyrus on granule cells and interneurons (Milner et al., 2000), with β1-ARs expressed in PV+ DG interneurons and β2-ARs in NPY+ DG interneurons (Cox et al., 2008). In CA3, β1-AR expression has been found on CCK+ interneurons (Cox et al., 2008). Although the functional effects of these receptors on interneurons is severely understudied, the distinct cellular expression profiles of these receptors suggest various modulatory roles of β-ARs in inhibitory transmission.

#### 3.2.2. Noradrenergic effects on cellular and synaptic properties

The effects of NA on cellular excitability are fairly modest, however NA typically hyperpolarizes and decreases the input resistance of CA3 pyramidal cells and dentate gyrus granule cells. Conversely, a small sub population also show depolarization and increased input resistance (Madison and Nicoll, 1986; Lacaille and Schwartzkroin, 1988; Ul Haq et al., 2012). These distinct effects are mediated by α- and β-ARs, respectively, with a β-AR mediated reduction in resting K^+^ conductance likely causing depolarization (Lacaille and Schwartzkroin, 1988). Since α2-AR activates Gi channels known to increase GIRK conductance (Sodickson and Bean, 1996; Lüscher et al., 1997), α2-AR activity may explain the hyperpolarizing effect of noradrenaline on hippocampal neurons, which reduces gain on excitatory glutamatergic transmission.

In the dentate gyrus, both inhibitory and excitatory hilar interneurons receive noradrenergic input (Milner and Bacon, 1989). In response to β-AR activation, both increase their firing rates, and inhibitory interneurons also show an attenuated sAHP (Bijak and Misgeld, 1995). α1-AR and α2-AR activation may attenuate discharges in inhibitory interneurons via hyperpolarization mechanisms (Bijak and Misgeld, 1995). The increased frequency of TTX-insensitive EPSPs and IPSPs observed in granule cells suggest that β-AR activation on both hilar interneuron types acts to increase presynaptic GABA and glutamate release. Interestingly, mixed α- and β-AR activation does not increase spontaneous discharge frequency, suggesting that α-AR activity can counteract β-AR effects. This may result from differing affinity to NA of each adrenoreceptor, meaning that the synaptic concentration of NA determines the direction of modulation via activation of distinct adrenoreceptor subtypes. Indeed lower concentrations have been found to be excitatory, whilst higher concentrations are inhibitory (Jurgens et al., 2005). This seems strange given that β1 receptors have lower affinity than α2-ARs (Ramos and Arnsten, 2007); however, this may be due to the greater proportion of β-ARs available for binding.

β-ARs enhance both NMDAR-dependent LTP and mossy fiber-LTP (Huang and Kandel, 1996; Gelinas, 2005). In the case of NMDAR-dependent LTP, this effect seems to occur both pre- and postsynaptically. Postsynaptically, β-ARs have been found to suppress sAHP currents, in turn increasing Ca^2+^ influx in granule cell and pyramidal cell synapses, reminiscent of M1 receptor effects (Madison and Nicoll, 1982; Haas and Konnerth, 1983; Haas and Rose, 1987; Pedarzani and Storm, 1993; Ul Haq et al., 2012). Furthermore, NA release during emotive stimuli have been found to facilitate the induction of LTP at pyramidal cell synapses by phosphorylation and subsequent trafficking of GluR1 containing AMPARs (Hu et al., 2007). It should be noted that in these LTP studies, adrenergic agonists were applied for ≥20 min, which may not correlate with NA levels following transient LC burst firing during novel or emotional stimuli. Burst activation of the LC has been shown to induce long-term heterosynaptic facilitation at perforant path synapses, reliant on β-AR activation (Walling and Harley, 2004). NA release can outlast LC firing in novel environments (McIntyre et al., 2002) and is regulated by presynaptic glutamate release onto LC terminals (Wang et al., 1992) such that extracellular NA release increases in response to perforant path high frequency stimulation (Bronzino et al., 2001).

Activation of presynaptically located β-ARs within the dentate gyrus at perforant path and mossy fiber terminals increases glutamate release (Lynch and Bliss, 1986), which is thought to be due to increased presynaptic calcium influx through L- and N-type calcium channels (Gray and Johnston, 1987). These presynaptic effects of β-ARs also enhance mossy fiber-LTP (Hopkins and Johnston, 1984, 1988). PKA activation via β-ARs is critical for both early and late phases of mossy fiber-LTP (Huang and Kandel, 1996) and pyramidal cell synapses (Gelinas, 2005; Gelinas et al., 2008). This process is likely to be dependent on phosphorylation of synapsin 1 and 2, both involved in the vesicle release process (Parfitt et al., 1991, 1992).

Together, the effects of β-ARs appear to enhance disynaptic entorhinal drive of CA3 via the dentate gyrus. Indeed, selective NA fiber lesions via 6-OHDA treatment reduce perforant path-DG LTP *in vivo* and *in vitro* (Bliss et al., 1983) and β-AR activation converts sub-threshold mossy fiber-LTP tetanus to supra-threshold (Huang and Kandel, 1996). Furthermore, this does not appear to affect recurrent CA3 synapses and is not influenced by GABA receptor activity (Huang and Kandel, 1996). It is interesting to note that other neuromodulators capable of raising intracellular cAMP, e.g., 5-HT, did not allow such LTP to be expressed, indicating the possible involvement of additional lateral signaling pathways.

Little research has addressed the role of α-ARs in the mossy fiber-CA3 microcircuit, however α1-ARs do reduce CA3 pyramidal cell sensitivity to mossy fiber transmission, without altering AMPAR sensitivity to glutamate (Scanziani et al., 1993) suggesting a presynaptic locus of action. However, α1-ARs can also enhance evoked NMDAR responses (Segal et al., 1991). Furthermore, α1-ARs appear to reduce basal release by decreasing presynaptic Ca^2+^ influx at mossy fiber-CA3 and recurrent CA3 synapses, but enhancing paired-pulse facilitation (Scanziani et al., 1993; Ul Haq et al., 2012). However, as with other neuromodulators, this effect may be indirect.

#### 3.2.3. Summary

- NA largely decreases cellular excitability in principal cells, but occasionally increases excitability.
- NA facilitates induction of Hebbian LTP.
- NA enables mPP-DG LTP.
- NA enhances mossy fiber synaptic transmission.
- NA ensures persistence of LTP.

### 3.3. Dopamine

#### 3.3.1. Dopamine release and receptors

Dopamine is released into the DG-CA3 microcircuit via projections from the Ventral Tegmental Area (VTA) and Substantia Nigra (SNc) (Gasbarri et al., 1994, 1997) and is associated with rewarding or novel stimuli. DA may also be released by noradrenergic fibers projecting from the locus coeruleus (LC) where it is stored in these afferents as a precursor (Smith and Greene, 2012) and DA antagonists can block the functional effects of LC stimulation in the hippocampus (Lemon and Manahan-Vaughan, 2012; Takeuchi et al., 2016). Furthermore, since DA reuptake transporters do not appear to be widely expressed in the hippocampus (Kwon et al., 2008), low levels of DA release could lead to extended periods of signaling.

DA receptors can be classified into two classes and five subtypes. D1 and D5 (D1-like) receptors that act to increase adenylate cyclase and cAMP, and D2-4 (D2-like) that decrease adenylate cyclase and cAMP (Missale et al., 1998). With developments in creating lines of transgenic mice, it has been easier to determine which cell types express particular subtypes. In the DG-CA3 microcircuit, D1 receptors are expressed on mature granule cells and CA3 interneurons (Gangarossa et al., 2012; Sariñana et al., 2014), while D5 is expressed on CA3 pyramidal cells and DG granule cells (Sariñana et al., 2014). Meanwhile, D2 receptors have been shown to be expressed strongly on hilar mossy cells and sparsely on CA3 interneurons, including PV+ basket cells and NPY+ ivy cells (Gangarossa et al., 2012; Etter and Krezel, 2014; Puighermanal et al., 2015). Overall, DA receptors are expressed more strongly in DG than CA3 (Ginsberg and Che, 2005), leading to the proposal that dopaminergic modulation of hippocampal function is primarily mediated by changes to DG function.

#### 3.3.2. Dopaminergic effects on cellular and synaptic properties

Evidence for effects of DA on cellular excitability in this circuit is limited. D1 receptor activity in dentate gyrus granule cells mediate enhancement of after-depolarization increasing the likelihood of bursts (Hamilton et al., 2010). Although typically inhibitory, D2 receptors increase excitability of hilar mossy cells through inhibition of SK3 channels (Etter and Krezel, 2014). This selective enhancement of mossy cell transmission may increase recurrent activity in the dentate gyrus, particularly along the dorso-ventral axis, which may be important for sequence learning.

Basal mossy fiber transmission is enhanced by D1/D5 receptor activation, and paired pulse facilitation is reduced (Kobayashi et al., 2006; Kobayashi and Suzuki, 2007) indicating a presynaptic action dependent on intracellular cAMP elevation (Tzounopoulos et al., 1998). However, Gangarossa et al. (2012) found no evidence of D1 receptors on mossy fiber boutons, possibly suggesting heterosynaptic spillover-mediated facilitation via presynaptic and extrasynaptic GABA-A, GABA-B, and kainate receptors (Vogt and Regehr, 2001; Chandler et al., 2003; Contractor et al., 2003; Ruiz et al., 2010).

Dopaminergic transmission may also influence inhibition in this circuit. Tonic enhancement of feedforward inhibition via D4 receptors has been reported in DG and CA1 interneurons (Romo-Parra et al., 2005; Rosen et al., 2015) although this may not be present in CA3 interneurons. However, D1/D5 activation has been found to enhance PV+ basket cell plasticity in CA3, which was further shown to be necessary for consolidation of new memories (Karunakaran et al., 2016).

A key component of the effect DA has on the DG-CA3 microcircuit is on plasticity in the mPP input to DG, primarily mediated through D1 receptors. Hamilton et al. (2010) demonstrated that increased dendritic excitability mediated by D1 activity facilitated induction of LTP in mPP-DG synapses, and Yang and Dani (2014) found that D1/D5 agonism alters STDP to convert pre-post intervals from depressing to potentiating, biasing the synapse toward potentiation overall and broadening the time window of STDP via inhibition of A-type K^+^ channels. Dopaminergic activity also appears to ensure the persistence of LTP. Sariñana et al. (2014) showed a deficit in late-LTP at this synapse in anesthetized D1 and D5 knockout mice, and D1/D5 activation can prevent low frequency stimulation induced depotentiation (Kulla and Manahan-Vaughan, 2000). This is similar to the effect of combined α1- and β-AR activation observed in CA1 (Katsuki et al., 1997). Furthermore, D1/D5 antagonism has been found to prevent the persistence of LTD at this synapse (Wiescholleck and Manahan-Vaughan, 2014) indicating that the effects of DA may be to promote persistence of plasticity regardless of sign. Collectively, DA-dependent synaptic plasticity facilitates the recruitment of larger ensembles of granule and pyramidal cells in context learning as shown by c-fos expression (Sariñana et al., 2014).

#### 3.3.3. Summary

- DA enhances mossy cell excitability.
- DA enables mPP-DG LTP induction.
- DA enhances mossy fiber synaptic transmission.
- DA facilitates inhibitory plasticity.
- DA ensures persistence of LTP.

### 3.4. Serotonin

#### 3.4.1. Serotonin release and receptors

The hippocampal formation receives serotonergic innervation from the median and dorsal raphe nuclei (MRN and DRN respectively) of the rostral serotonergic raphe system (Törk, 1990; Gulyás et al., 1999; Leranth and Hajszan, 2007). Axons of the MRN 5-HT neurons (M fibers) are thick with large irregular varicosities, and make classical synaptic connections. These innervate the hippocampus on its entire dorso-ventral axis, terminating in the subiculum, stratum lacunosum-moleculare in CA1-CA3, stratum radiatum and oriens in CA2 and CA3, and in the polymorphic layer of the dentate gyrus. 5-HT neurons in the DRN send an extensively ramified, diffuse system of thin axons with many spindle-like varicosities (D-fibers) that mostly innervate the stratum lacunosum-moleculare and the polymorphic layer of the dentate gyrus. Delivery of 5-HT to the hippocampus depends on the firing of serotonergic cells, which have been found to fire at a constant 3 Hz rate during quiet wake, but increase two to five-fold during repetitive motor activity, e.g., running, and fall largely silent during REM sleep and in the presence of novel attention-orienting stimuli (Jacobs and Fornal, 1999), however local raphe circuits may interact to deliver 5-HT to regions of the brain at different volumes.

There are currently at least 14 known distinct subtypes of 5-HT receptor, of which all are G protein coupled except 5-HT3 (Barnes and Sharp, 1999; Millan et al., 2008; Berumen et al., 2012), with many of them found to be functionally expressed in the DG-CA3 microcircuit. 5-HT1 receptor types couple to Gi/o proteins that inhibit adenylate cyclase and cAMP synthesis, and one important subtype (5-HT1A) is highly expressed somatodendritically on CA3 pyramidal cells, dentate granule cells, and PV+ interneurons (Pazos et al., 1985; Andrade et al., 1986; Chalmers and Watson, 1991; Aznar et al., 2003). 5-HT2A and 5-HT2C receptors couple via Gq/11 to the IP3/PLC signaling pathway, and have been found on granule cells, pyramidal cells, and interneurons in DG and CA3 (Li et al., 2004; Bombardi, 2012). 5-HT4 and 5-HT7 receptors couple to Gs proteins and promote cAMP synthesis, with both expressed on granule cells and pyramidal cells, and 5-HT7 receptors specifically on CA3 pyramidal cells (Vilaró et al., 1996, 2005; Suwa et al., 2014); however, expression of 5-HT4 decreases during memory consolidation (Manuel-Apolinar et al., 2005). Finally, 5-HT3 receptors are ligand gated non-selective cation channels that belong to the same superfamily as nicotinic, GABA, and glycine receptors, and have been found on CCK+ basket cells, providing a fast depolarizing inward current (Freund et al., 1990; McMahon and Kauer, 1997; Gulyás et al., 1999).

#### 3.4.2. Serotonergic effects on cellular and synaptic properties

5-HT1A receptors mediate a strong hyperpolarizing effect of 5-HT on granule cells and pyramidal cells through activation of GIRK channels (Andrade and Nicoll, 1987; Beck et al., 1992; Okuhara and Beck, 1994; Piguet and Galvan, 1994; Twarkowski et al., 2016; Ul Haq et al., 2016), reducing firing frequency, and dampening gamma oscillations (Twarkowski et al., 2016). This hyperpolarization is opposed by activation of 5-HT4 receptors on pyramidal and granule cells, and 5-HT7 receptors on pyramidal cells that inhibit sAHP currents to increase firing frequency (Bacon and Beck, 2000). Collectively, findings from *in vitro* pharmacology studies suggest that the net effect of 5-HT in CA3 is widely inhibitory, but 5-HT also influences neuronal gain whereby weak excitatory stimuli are suppressed and strong excitatory stimuli are enhanced (Andrade et al., 1986; Beck et al., 1992; Villani and Johnston, 1993). In the dentate gyrus however, the distribution of 5-HT1A receptors along granule cell dendrites induces a shunting inhibition that blocks lateral PP over medial PP input (Nozaki et al., 2016). It is important to note that the balance of hyperpolarizing and depolarizing effects of 5-HT will depend on the local concentration of 5-HT available given the varying density and affinity of receptors.

The effects of 5-HT on synaptic transmission have been somewhat contradictory. Some studies have found that 5-HT prevents the induction of NMDAR dependent LTP (Villani and Johnston, 1993) due to the hyperpolarizing effect on pyramidal cells. However, when administered at concentrations that do not hyperpolarize cells 5-HT4 receptor activation is found to prevent mPP-DG LTD and curtail established LTP (Twarkowski et al., 2016). Furthermore, release of endogenous 5-HT via MDMA application has been found to facilitate LTP induction (Mlinar et al., 2015). 5-HT2 receptors have also been found to mediate synaptic facilitation via a PLC pathway by pre- or postsynaptic mechanisms, but this may also be occluded by an increase in GABAergic synaptic activity that diffuses extrasynaptically to tonically inhibit this effect (Zhang and Stackman, 2015). Altogether, it is highly unclear what the likely effect of endogenously released 5-HT under naturalistic conditions is likely to have on the induction of long term plasticity.

At the mossy fiber synapse, 5-HT4 activation has been found to prevent both LTP and LTD at 5-HT concentrations that do not hyperpolarize cells. Earlier studies also found that 5-HT3 receptor activity prevents mossy fiber LTP (Maeda et al., 1994), which is likely due to recruitment of CCK+ basket cells that fire asynchronously to tonically inhibit CA3 pyramidal cells. Furthermore, 5-HT1A and 5-HT4 receptors modulate mossy fiber synaptic transmission, as the NMDAR-mediated EPSC components are enhanced by 5-HT4 activity, and are concurrently mildly suppressed by a longer lasting 5-HT1A effect (Kobayashi et al., 2008).

#### 3.4.3. Summary

- 5-HT is principally hyperpolarizing.
- 5-HT can both enhance or suppresses Hebbian LTP.
- 5-HT suppresses lPP-DG synapses.
- 5-HT can both enhance or suppress mossy fiber transmission.

## 4. Implications for theories of neuromodulation and memory encoding and retrieval

Theories and computational models of memory encoding and retrieval in the hippocampus often compromise between incorporating features of observed behavior (top-down) either as memory task performance or network dynamics, and circuit dynamics at the cellular level (bottom-up) in terms of excitability of cellular membranes and synaptic plasticity. In this review we have broadly outlined results on the latter components of these theories. The net effect of neuromodulators is often difficult to predict as receptor subtypes may have apparently opposing effects, or may take place over distinct time scales. As such, the net effects and implications for encoding and retrieval are ideally placed for investigation through computational modeling. In this section, we review computational models of DG-CA3 network function in encoding and retrieval that have incorporated features of neuromodulation, and discuss how these models can be adapted and extended to include multiple neuromodulatory systems or incorporate further features of excitability and plasticity that have received little theoretical treatment. In particular, since cholinergic neuromodulation has received the most extensive consideration, but shares many mechanisms of action with other neuromodulatory systems, it will be used as a starting point to suggest how other neuromodulators may influence encoding and retrieval through changes to excitability and plasticity.

### 4.1. Encoding: learning conditions

Several theories argue that ACh is essential for learning new informations patterns through a variety of mechanisms. One family of theories inspired by Marr's theory of archicortex (Marr, 1971) propose that ACh selectively enhances information flow through DG-CA3 to decorrelate and sparsify entorhinal information, reducing interference during encoding (Hasselmo, 2006; Hummos et al., 2014). However, others have proposed that through enhancement of the power of theta and gamma rhythms, ACh opens tightly controlled windows for spike timing-dependent plasticity (STDP) driven by either afferent input to form associations (Kunec et al., 2005; Bush et al., 2010; Jochems and Yoshida, 2015). Furthermore, ACh also increases excitability which favors pre- and postsynaptic burst firing necessary to activate STDP (Bush et al., 2010; Jochems and Yoshida, 2015; Saravanan et al., 2015).

NA may play a similar role as ACh by creating an environment ripe for encoding through the actions of β-ARs, since they similarly suppress sAHP currents that lowers the induction threshold for NMDAR-dependent plasticity and enhance excitability in a subpopulation of neurons (Madison and Nicoll, 1982; Pedarzani and Storm, 1993; Gelinas, 2005; Ul Haq et al., 2012). However, in contrast with ACh, this enhancement in excitability is not as widespread providing a more conservative encoding system. Likewise, although 5-HT may help to induce synaptic plasticity through 5-HT2, 5-HT4, or 5-HT7 receptors, due to common signaling pathways, some coordinated disinhibition or relief from strong hyperpolarization may need to take place to permit this, making any learning via 5-HT even more conservative. DA on the other hand, may have a similarly liberal encoding system to ACh, albeit restricted to dentate gyrus granule cells where expression of DA receptors is greatest (Hamilton et al., 2010), and may confer the ability to encode delayed associations by broadening the STDP window to include potentiating post-pre pairs (Yang and Dani, 2014).

Much of our knowledge about synaptic plasticity is inferred from studies using prolonged high frequency stimulation, although STDP is thought to be a more biologically realistic stimulation paradigm. Since the key trigger for synaptic plasticity is calcium influx into postsynaptic spines, understanding how neuromodulators gate this process via control of nanodomain calcium concentration to trigger common calcium dependent signaling pathways (Buchanan et al., 2010; Giessel and Sabatini, 2010; Griffith et al., 2016; Tigaret et al., 2016) will be critical to provide a better understanding of encoding under biologically relevant conditions.

### 4.2. Encoding: novelty detection

The hippocampus favors the encoding of neuronal ensembles in novel situations and neuromodulators are proposed to achieve this encoding by reducing the threshold for novelty detection. A recent model proposed a mechanism in which novelty is detected via the relative timing of DG and CA3 firing, i.e., if CA3 fires before DG then the stimulus is familiar so no learning should take place (Nolan et al., 2011). In this scenario, ACh and DA lower the threshold for novelty detection by selectively enhancing the EC-DG-CA3 pathway, thereby speeding up transmission and triggering encoding more often (Nolan et al., 2011). This theory is supported by the finding that CA3 pyramidal cells can activate a disinhibitory circuit via the lateral septum to promote DA release from the VTA (Luo et al., 2011). Similarly, earlier models of novelty detection proposed that new patterns would be filtered out by the dentate gyrus, reducing CA3 firing and promoting ACh release by disinhibiting the medial septum (Meeter et al., 2004). Conversely, when 5-HT is introduced, the hyperpolarizing effect of 5-HT1ARs dominates information flow in this circuit, and raises the novelty detection threshold (Meeter et al., 2006). Finally, NA may increase or decrease novelty detection thresholds due to the mixture of hyperpolarization and enhancement of the EC-DG-CA3 pathway.

### 4.3. Information flow

It is useful to consider how the effects of neuromodulators on excitability and plasticity relate to gain modulation and signal-to-noise ratio through the combined action of GIRK enhancement and sAHP suppression. Each of the neuromodulators act through these potassium channels to regulate excitability and this balance is important for understanding how information flows through this circuit in different configurations. Through inward rectification, GIRK channel activation provides shunting inhibition at rest that attenuates toward threshold, while sAHP current suppression increases firing frequency. Collectively, these effects can be predicted to suppress weak synaptic input and increase the gain on strong synaptic input, further increasing the likelihood of bursts with strong synaptic input. This effectively increases signal far above noise, and may facilitate propagation of coordinated recurrent activity.

In contrast, ACh primarily suppresses sAHP currents increasing the likelihood of spontaneous spiking across excitatory and inhibitory cells thereby increasing both signal and noise, which may effectively drown out competing streams of information with noise. This may be a particular mechanism through which strongly fluctuating afferent input, e.g., mossy fibers or synchronous perforant path input, are favored over weakly fluctuating inputs e.g., recurrent or asynchronous perforant path input. For other neuromodulatory systems, 5-HT may increase signal with respect to noise more weakly than NA whereas DA may act similarly to NA, but with greater restriction to the dentate gyrus as opposed to CA3 due to higher expression of D1 receptors in dentate gyrus.

### 4.4. Autoassociation (pattern completion)

In theories proposing that ACh supports pattern separation, it is often also proposed to suppress pattern completion due to presynaptic inhibition of recurrent collaterals (Hasselmo, 2006; Hummos et al., 2014). However, when incorporating the effects of ACh on intrinsic excitability, pattern completion in CA3 is actually more robust due to intrinsically driven bursting, despite suppressed recurrent synapses (Jochems and Yoshida, 2015). Furthermore, distal inhibition keeps burst propagation local, allowing pattern completion amongst small networks (Saravanan et al., 2015). Theta-driven models of retrieval in which encoding and retrieval take place within one cycle of the theta rhythm, propose that ACh enhances the theta rhythm and for half a cycle disinhibits OLM cells that inhibit perforant path inputs to CA3 pyramidal cells and render mossy fiber cued autoassociative dynamics dominant (Kunec et al., 2005).

Alternative theories propose that pattern completion takes place in the dentate gyrus, when mossy cells are activated to provide feedback excitation (Lisman, 1999; Lisman and Otmakhova, 2001). Granule cells have been shown to participate in pattern completion (Leutgeb et al., 2007), and DA may have a role in converting the dentate gyrus from pattern separation to pattern completion mode by increasing the excitability of mossy cells via D2 receptors (Etter and Krezel, 2014).

### 4.5. Retrieval: heteroassociation (sequence completion)

Some theories argue that the CA3 network plays a role in sequence completion (Lisman, 1999), and that sharp wave ripples, replay, preplay and phase precession are phenomena resulting from sequence completion. Within this context, ACh is proposed to suppress sequence completion since it suppresses recurrent collaterals necessary for fast replay, and suppresses AHP currents necessary to propagate signals unidirectionally with bidirectional synapses (Saravanan et al., 2015).

Furthermore, *in vitro* sharp wave ripples are bidirectionally influenced by NA via hyperpolarization of CA3 pyramidal cells and activation of excitability-enhancing plasticity-inducing β-ARs, meaning the spatial distribution of ARs may be important in determining the precision of both encoding and retrieval (Ul Haq et al., 2012). In contrast, ACh shifts ripple frequencies to gamma frequencies (Fischer et al., 2014), indicative of a switch from retrieval to encoding states. The effect of NA in this case is more similar to 5-HT, which largely suppresses sharp wave ripples through hyperpolarization induced by 5-HT1A receptors (Ul Haq et al., 2016). DA enhances sharp wave ripple replay, partly through plasticity of interneuron synapses (Miyawaki et al., 2014; Karunakaran et al., 2016). This suggests DA may push the DG-CA3 system toward a retrieval dominated state of processing.

### 4.6. Conclusions and future directions

Modulation of excitability and plasticity within the DG-CA3 microcircuit by the neuromodulators described here is likely to alter its function. In this review we have outlined known mechanisms through which alterations can occur, and how these may relate to encoding and retrieval. There are many similarities between each type of neuromodulator and as such their effects may well take place in different regions of the same “state space.” Here we have proposed a system in which each neuromodulator may favor encoding (ACh and NA) or retrieval (DA and 5-HT), with greater likelihood of false positives (ACh and DA) in a liberal state, and false negatives (NA and 5-HT) in a conservative state. These effects may take place through altering subthreshold integration and filtering of inputs via distinct potassium channel conductances, and selective augmentation of afferent synaptic pathways and synaptic noise.

Certain aspects of neuromodulatory effects have yet to be addressed in the dentate-CA3 microcircuit, for example, the shared role of NA and DA in ensuring the persistence of LTP at the mPP-DG synapse (Straube and Frey, 2003; Hamilton et al., 2010; Sariñana et al., 2014). This may be necessary to associate ensembles in sequence that are delayed over a longer period of time, or could be necessary to cue re-encoding and consolidation during subsequent retrieval. Likewise the effects of neuromodulators on interneuron excitability, plasticity, and function are rarely considered but are likely to be very important. In many theories, inhibitory networks are designed to produce a particular set of dynamics, introducing a heavy bias. Since plasticity in inhibitory networks has been shown to be sufficient to support encoding and retrieval in recurrent networks by fine tuning of excitatory-inhibitory balance (Vogels et al., 2011), a greater understanding of the role of neuromodulators within this framework could reduce such bias. For example, the recent finding that inhibitory plasticity of PV+ basket cells is enhanced by D1 receptors during memory consolidation (Karunakaran et al., 2016) may be through the enhancement of gamma power that allows repeated efficient retrieval of sequences.

Finally it is necessary to understand the spatio-temporal release profile of neuromodulators under physiological conditions to assess the validity of these theories. Much of our current knowledge is through pharmacological manipulation of excitability and plasticity *in vitro*, which may be well suited to understanding the steady state effect of neuromodulators, but is not so informative of dynamic neuromodulatory systems. For example, Rosen et al. (2015) used optogenetic stimulation of VTA and SNc terminals in the hippocampus to show biphasic effects of DA receptors in CA1, with tonic release increasing inhibitory drive by enhancing Schaffer Collateral excitation of PV+ basket cells via D4 receptors and phasic release increasing excitatory drive by enhancing Schaffer Collateral excitation of pyramidal cells via D1/D5 receptors. Previously, pharmacological activation of these receptors had been unable to uncover this biphasic effect. Such control may also be expected to be observed in the dentate gyrus given a similar distribution of DA receptors.

It is very likely that control of the DG-CA3 circuit by other neuromodulatory systems will also be sensitive to neuromodulator release profile. Varga et al. (2009) demonstrated that median raphe nuclei projections to the CA1 and CA3 rapidly recruit a powerful inhibitory network via mixed serotonergic-glutamatergic release that activates postsynaptic 5-HT3 and glutamate receptors. Similarly, Grybko et al. (2011) showed that low frequency electrical stimulation of cholinergic fibers elicited fast nicotinic mediated depolarization of CA3 pyramidal cells and interneurons, which are typically occluded by rapid desensitization of α7 receptors with bath application of cholinergic agonists. The balance between muscarinic and nicotinic control of circuit function may therefore vary with the pattern of cholinergic delivery by the medial septum. High frequency locus coeruleus fiber stimulation has also been shown to modulate long-term depression and potentiation at mPP-DG synapses via β-AR activation, which may indicate that LC bursts during emotive, arousing, or novel stimuli “opens the gate” for mPP-DG plasticity (Hansen and Manahan-Vaughan, 2015a,b), and may contribute to any role the dentate gyrus performs in novelty detection.

Furthermore, neuromodulatory systems are known to interact such that co-release or sequentially co-ordinated release may be highly relevant to their circuit impact. For example, feedforward and feedback control of neuromodulatory systems between the habenula and the hippocampus has been shown with regards to serotonergic downregulation and cholinergic upregulation of the hippocampal theta rhythm (Aizawa et al., 2013; Goutagny et al., 2013), and 5-HT4 agonists delivered to the medial septum can increase ACh release to the hippocampus (Mohler et al., 2007). In such cases similar effects may saturate or augment each other, or opposing effects could cancel each other out. If there is a coding system of uncertainty, novelty, and salience related to these neuromodulators then these are likely represented through a dynamic neuromodulatory profile, whose dissection remains a fascinating topic of investigation.

## Author contributions

All authors listed, have made substantial, direct and intellectual contribution to the work, and approved it for publication.

## Funding

LP, CT, and JM supported by Wellcome Trust. TB, CT, and JM supported by Biotechnology and Biological Science Research Council.

### Conflict of interest statement

The authors declare that the research was conducted in the absence of any commercial or financial relationships that could be construed as a potential conflict of interest.

## References

[B1] Acsády L. Kamondi A. Sík A. Freund T. F. Buzsáki G. (1998). GABAergic cells are the major postsynaptic targets of mossy fibers in the rat hippocampus. J. Neurosci. 18, 3386–3403. 9547246 10.1523/JNEUROSCI.18-09-03386.1998 PMC6792657

[B2] Ahumada J. de Sevilla D. F. Couve A. Buño W. Fuenzalida M. (2013). Long-term depression of inhibitory synaptic transmission induced by spike-timing dependent plasticity requires coactivation of endocannabinoid and muscarinic receptors. Hippocampus 23, 1439–1452. 10.1002/hipo.22196 23966210

[B3] Aizawa H. Yanagihara S. Kobayashi M. Niisato K. Takakawa T. Harukuni R.. (2013). The synchronous activity of lateral habenular neurons is essential for regulating hippocampal theta oscillation. J. Neurosci. 33, 8909–8921. 10.1523/JNEUROSCI.4369-12.2013 23678132 PMC6618841

[B4] Akam T. Oren I. Mantoan L. Ferenczi E. Kullmann D. M. (2012). Oscillatory dynamics in the hippocampus support dentate gyrus-CA3 coupling. Nat. Neurosci. 15, 763–768. 10.1038/nn.3081 22466505 PMC3378654

[B5] Alkondon M. Albuquerque E. X. (2001). Nicotinic acetylcholine receptor α7 and α4β2 subtypes differentially control GABAergic input to CA1 neurons in rat hippocampus. J. Neurophysiol. 86, 3043–3055. Available online at: http://jn.physiology.org/content/86/6/3043 11731559 10.1152/jn.2001.86.6.3043

[B6] Andrade R. Malenka R. C. Nicoll R. A. (1986). A G-protein couples serotonin and GABAB receptors to the same channels in hippocampus. Science 234, 1261–1265. 10.1126/science.2430334 2430334

[B7] Andrade R. Nicoll R. A. (1987). Pharmacologically distinct actions of serotonin on single pyramidal neurones of the rat hippocampus recorded *in vitro*. J. Physiol. 394, 99–124. 10.1113/jphysiol.1987.sp016862 3443977 PMC1191953

[B8] Aznar S. Qian Z. Shah R. Rahbek B. Knudsen G. M. (2003). The 5-HT1A serotonin receptor is located on calbindin- and parvalbumin-containing neurons in the rat brain. Brain Res. 959, 58–67. 10.1016/S0006-8993(02)03727-7 12480158

[B9] Bacon W. L. Beck S. G. (2000). 5-Hydroxytryptamine7 receptor activation decreases slow afterhyperpolarization amplitude in CA3 hippocampal pyramidal cells. J. Pharmacol. Exp. Ther. 294, 672–679. Available online at: http://jpet.aspetjournals.org/content/294/2/672 10900247

[B10] Barnes N. M. Sharp T. (1999). A review of central 5-HT receptors and their function. Neuropharmacology 38, 1083–1152. 10.1016/S0028-3908(99)00010-6 10462127

[B11] Beck S. G. Choi K. C. List T. J. (1992). Comparison of 5-hydroxytryptamine1A-mediated hyperpolarization in CA1 and CA3 hippocampal pyramidal cells. J. Pharmacol. Exp. Ther. 263, 350–359. 1403796

[B12] Berumen L. C. Rodriguez A. Miledi R. Garcia-Alcocer G. (2012). Serotonin receptors in hippocampus. ScientificWorldJournal 2012:823493. 10.1100/2012/823493 22629209 PMC3353568

[B13] Bijak M. Misgeld U. (1995). Adrenergic modulation of hilar neuron activity and granule cell inhibition in the guinea-pig hippocampal slice. Neuroscience 67, 541–550. 10.1016/0306-4522(95)00086-X 7675185

[B14] Bliss T. V. P. Goddard G. V. Riives M. (1983). Reduction of long-term potentiation in the dentate gyrus of the rat following selective depletion of monoamines. J. Physiol. 334, 475–491. 10.1113/jphysiol.1983.sp014507 6864566 PMC1197327

[B15] Bombardi C. (2012). Neuronal localization of 5-HT2A receptor immunoreactivity in the rat hippocampal region. Brain Res. Bull. 87, 259–273. 10.1016/j.brainresbull.2011.11.006 22119732

[B16] Brandalise F. Gerber U. (2014). Mossy fiber-evoked subthreshold responses induce timing-dependent plasticity at hippocampal CA3 recurrent synapses. Proc. Natl. Acad. Sci. U.S.A. 111, 4303–4308. 10.1073/pnas.1317667111 24550458 PMC3964078

[B17] Bronzino J. D. Kehoe P. Mallinson K. Fortin D. A. (2001). Increased extracellular release of hippocampal NE is associated with tetanization of the medial perforant pathway in the freely moving adult male rat. Hippocampus 11, 423–429. 10.1002/hipo.1057 11530847

[B18] Buchanan K. A. Petrovic M. M. Chamberlain S. E. L. Marrion N. V. Mellor J. R. (2010). Facilitation of long-term potentiation by muscarinic M1 receptors is mediated by inhibition of SK channels. Neuron 68, 948–963. 10.1016/j.neuron.2010.11.018 21145007 PMC3003154

[B19] Bush D. Philippides A. Husbands P. O'Shea M. (2010). Dual coding with STDP in a spiking recurrent neural network model of the hippocampus. PLoS Comput. Biol. 6:e1000839. 10.1371/journal.pcbi.1000839 20617201 PMC2895637

[B20] Chalmers D. T. Watson S. J. (1991). Comparative anatomical distribution of 5-HT1A receptor mRNA and 5-HTIA binding in rat brain – a combined *in situ* hybridisation/*in vitro* receptor autoradiographic study. Brain Res. 561, 51–60. 10.1016/0006-8993(91)90748-K 1797349

[B21] Chandler K. E. Princivalle A. P. Fabian-Fine R. Bowery N. G. Kullmann D. M. Walker M. C. (2003). Plasticity of GABA_B_ receptor-mediated heterosynaptic interactions at mossy fibers after status epilepticus. J. Neurosci. 23, 11382–11391. Available online at: http://www.jneurosci.org/content/23/36/11382 14673002 10.1523/JNEUROSCI.23-36-11382.2003 PMC6740526

[B22] Cheng Q. Yakel J. L. (2014). Presynaptic α7 nicotinic acetylcholine receptors enhance hippocampal mossy fiber glutamatergic transmission via PKA activation. J. Neurosci. 34, 124–133. 10.1523/JNEUROSCI.2973-13.2014 24381273 PMC3866480

[B23] Chiang P. H. Yeh W. C. Lee C. T. Weng J. Y. Huang Y. Y. Lien C. C. (2010). M1-like muscarinic acetylcholine receptors regulate fast-spiking interneuron excitability in rat dentate gyrus. Neuroscience 169, 39–51. 10.1016/j.neuroscience.2010.04.051 20433901

[B24] Contractor A. Sailer A. W. Darstein M. Maron C. Xu J. Swanson G. T.. (2003). Loss of kainate receptor-mediated heterosynaptic facilitation of mossy-fiber synapses in KA2^−/−^ mice. J. Neurosci. 23, 422–429. Available online at: http://www.jneurosci.org/content/23/2/422 12533602 10.1523/JNEUROSCI.23-02-00422.2003 PMC6741894

[B25] Cox D. J. Racca C. LeBeau F. E. N. (2008). alpha-adrenergic receptors are differentially expressed in distinct interneuron subtypes in the rat hippocampus. J. Comp. Neurol. 509, 551–565. 10.1002/cne.21758 18546278

[B26] Danielson N. B. Kaifosh P. Zaremba J. D. Lovett-Barron M. Tsai J. Denny C. A.. (2016). Distinct contribution of adult-born hippocampal granule cells to context encoding. Neuron 90, 101–112. 10.1016/j.neuron.2016.02.019 26971949 PMC4962695

[B27] Dasari S. Gulledge A. T. (2011). M1 and M4 receptors modulate hippocampal pyramidal neurons. J. Neurophysiol. 105, 779–792. 10.1152/jn.00686.2010 21160001 PMC3059175

[B28] Domínguez S. Fernández de Sevilla D. Buño W. (2014). Postsynaptic activity reverses the sign of the acetylcholine-induced long-term plasticity of GABAA inhibition. Proc. Natl. Acad. Sci. U.S.A. 111, E2741–E2750. 10.1073/pnas.1321777111 24938789 PMC4084432

[B29] Domínguez S. Fernández de Sevilla D. Buño W. (2015). Acetylcholine facilitates a depolarization-induced enhancement of inhibition in rat CA1 pyramidal neurons. Cereb. Cortex. [Epub ahead of print]. 10.1093/cercor/bhv276 26620268

[B30] Donato F. Belluco Rompani S. Caroni P. (2013). Parvalbumin-expressing basket-cell network plasticity induced by experience regulates adult learning. Nature 504, 272–276. 10.1038/nature12866 24336286

[B31] Etter G. Krezel W. (2014). Dopamine D2 receptor controls hilar mossy cells excitability. Hippocampus 24, 725–732. 10.1002/hipo.22280 24753432

[B32] Fabian-Fine R. Skehel P. Errington M. L. Davies H. A. Sher E. Stewart M. G.. (2001). Ultrastructural distribution of the α7 nicotinic acetylcholine receptor subunit in rat hippocampus. J. Neurosci. 21, 7993–8003. Available online at: http://www.jneurosci.org/content/21/20/7993 11588172 10.1523/JNEUROSCI.21-20-07993.2001 PMC6763871

[B33] Fischer V. Both M. Draguhn A. Egorov A. V. (2014). Choline-mediated modulation of hippocampal sharp wave-ripple complexes *in vitro*. J. Neurochem. 129, 792–805. 10.1111/jnc.12693 24673342

[B34] Frazier C. J. Strowbridge B. W. Papke R. L. (2003). Nicotinic receptors on local circuit neurons in dentate gyrus : a potential role in regulation of granule cell excitability nicotinic receptors on local circuit neurons in dentate gyrus : a potential role in regulation of granule cell excitability. J. Neurophysiol. 89, 3018–3028. 10.1152/jn.01036.2002 12611982

[B35] Freund T. F. Gulyás A. I. Acsády L. Görcs T. Tóth K. (1990). Serotonergic control of the hippocampus via local inhibitory interneurons. Proc. Natl. Acad. Sci. U.S.A. 87, 8501–8505. 10.1073/pnas.87.21.8501 1700433 PMC54984

[B36] Fuhs M. C. Touretzky D. S. (2000). Synaptic learning models of map separation in the hippocampus. Neurocomputing 32–33, 379–384. 10.1016/S0925-2312(00)00189-2

[B37] Fukudome Y. Ohno-Shosaku T. Matsui M. Omori Y. Fukaya M. Tsubokawa H.. (2004). Two distinct classes of muscarinic action on hippocampal inhibitory synapses: M2-mediated direct suppression and M1/M3-mediated indirect suppression through. Eur. J. Neurosci. 19, 2682–2692. 10.1111/j.0953-816X.2004.03384.x 15147302

[B38] Gahring L. C. Persiyanov K. Dunn D. Weiss R. Meyer E. L. Rogers S. W. (2004). Mouse strain-specific nicotinic acetylcholine receptor expression by inhibitory interneurons and astrocytes in the dorsal hippocampus. J. Comp. Neurol. 468, 334–346. 10.1002/cne.10943 14681929

[B39] Gahring L. C. Rogers S. W. (2008). Nicotinic acetylcholine receptor expression in the hippocampus of 27 mouse strains reveals novel inhibitory circuitry. Hippocampus 18, 737–749. 10.1002/hipo.20430 18446824 PMC2792088

[B40] Gangarossa G. Longueville S. De Bundel D. Perroy J. Hervé D. Girault J. A.. (2012). Characterization of dopamine D1 and D2 receptor-expressing neurons in the mouse hippocampus. Hippocampus 22, 2199–2207. 10.1002/hipo.22044 22777829

[B41] Gasbarri A. Sulli A. Packard M. G. (1997). The dopaminergic mesencephalic projections to the hippocampal formation in the rat. Prog. Neuropsychopharmacol. Biol. Psychiatry 21, 1–22. 9075256 10.1016/s0278-5846(96)00157-1

[B42] Gasbarri A. Verney C. Innocenzi R. Campana E. Pacitti C. (1994). Mesolimbic dopaminergic neurons innervating the hippocampal formation in the rat: a combined retrograde tracing and immunohistochemical study. Brain Res. 668, 71–79. 10.1016/0006-8993(94)90512-6 7704620

[B43] Gelinas J. N. (2005). Beta-adrenergic receptor activation facilitates induction of a protein synthesis-dependent late phase of long-term potentiation. J. Neurosci. 25, 3294–3303. 10.1523/JNEUROSCI.4175-04.2005 15800184 PMC6724894

[B44] Gelinas J. N. Tenorio G. Lemon N. Abel T. Nguyen P. V. (2008). Beta-adrenergic receptor activation during distinct patterns of stimulation critically modulates the PKA-dependence of LTP in the mouse hippocampus. Learn. Mem. 15, 281–289. 10.1101/lm.829208 18441285 PMC2364601

[B45] Giessel A. J. Sabatini B. L. (2010). M1 muscarinic receptors boost synaptic potentials and calcium influx in dendritic spines by inhibiting postsynaptic SK channels. Neuron 68, 936–947. 10.1016/j.neuron.2010.09.004 21145006 PMC3052967

[B46] Ginsberg S. D. Che S. (2005). Expression profile analysis within the human hippocampus: comparison of CA1 and CA3 pyramidal neurons. J. Comp. Neurol. 487, 107–118. 10.1002/cne.20535 15861457

[B47] Giocomo L. M. Hasselmo M. E. (2005). Nicotinic modulation of glutamatergic synaptic transmission in region CA3 of the hippocampus. Eur. J. Neurosci. 22, 1349–1356. 10.1111/j.1460-9568.2005.04316.x 16190890

[B48] Goutagny R. Loureiro M. Jackson J. Chaumont J. Williams S. Isope P.. (2013). Interactions between the lateral habenula and the hippocampus: implication for spatial memory processes. Neuropsychopharmacology 38, 1–9. 10.1038/npp.2013.142 23736315 PMC3799061

[B49] Gray R. Johnston D. (1987). Noradrenaline and β-adrenoceptor agonists increase activity of voltage-dependent calcium channels in hippocampal neurons. Nature 327, 620–622. 10.1038/327620a0 2439913

[B50] Griffith T. Tsaneva-Atanasova K. Mellor J. R. (2016). Control of Ca2+ influx and calmodulin activation by SK-channels in dendritic spines. PLoS Comput. Biol. 12:e1004949. 10.1371/journal.pcbi.1004949 27232631 PMC4883788

[B51] Grishin A. A. Benquet P. Gerber U. (2005). Muscarinic receptor stimulation reduces NMDA responses in CA3 hippocampal pyramidal cells via Ca2+-dependent activation of tyrosine phosphatase. Neuropharmacology 49, 328–337. 10.1016/j.neuropharm.2005.03.019 15993905

[B52] Grishin A. A. Gee C. E. Gerber U. Benquet P. (2004). Differential calcium-dependent modulation of NMDA currents in CA1 and CA3 hippocampal pyramidal cells. J. Neurosci. 24, 350–355. 10.1523/JNEUROSCI.4933-03.2004 14724233 PMC6729976

[B53] Grybko M. Sharma G. Vijayaraghavan S. (2010). Functional distribution of nicotinic receptors in CA3 region of the hippocampus. J. Mol. Neurosci. 40, 114–120. 10.1007/s12031-009-9266-8 19693709 PMC2871704

[B54] Grybko M. J. Hahm E. T. Perrine W. Parnes J. A. Chick W. S. Sharma G.. (2011). A transgenic mouse model reveals fast nicotinic transmission in hippocampal pyramidal neurons. Eur. J. Neurosci. 33, 1786–1798. 10.1111/j.1460-9568.2011.07671.x 21501254 PMC3095690

[B55] Gulyás A. I. Acsády L. Freund T. F. (1999). Structural basis of the cholinergic and serotonergic modulation of GABAergic neurons in the hippocampus. Neurochem. Int. 34, 359–372. 10.1016/S0197-0186(99)00041-8 10397363

[B56] Gundlfinger A. Breustedt J. Sullivan D. Schmitz D. (2010). Natural spike trains trigger short- and long-lasting dynamics at hippocampal mossy fiber synapses in rodents. PLoS ONE 5:e9961. 10.1371/journal.pone.0009961 20376354 PMC2848597

[B57] Gundlfinger A. Leibold C. Gebert K. Moisel M. Schmitz D. Kempter R. (2007). Differential modulation of short-term synaptic dynamics by long-term potentiation at mouse hippocampal mossy fibre synapses. J. Physiol. 585(Pt 3), 853–865. 10.1113/jphysiol.2007.143925 17962326 PMC2375525

[B58] Guo N. N. Li B. M. (2007). Cellular and subcellular distributions of alpha1- and alpha2-Adrenoceptors in the CA1 and CA3 regions of the rat hippocampus. Neuroscience 146, 298–305. 10.1016/j.neuroscience.2007.01.013 17337326

[B59] Guzman S. J. Schlögl A. Frotscher M. Jonas P. (2016). Synaptic mechanisms of pattern completion in the hippocampal ca3 network. Science 353, 1117–1123. 10.1126/science.aaf1836 27609885

[B60] Haas H. L. Konnerth A. (1983). Histamine and noradrenaline decrease calcium-activated potassium conductance in hippocampal pyramidal cells. Nature 302, 432–434. 10.1038/302432a0 6300681

[B61] Haas H. L. Rose G. M. (1987). Noradrenaline blocks potassium conductance in rat dentate gyrus cells *in vitro*. Neurosci. Lett. 78, 171–174. 10.1016/0304-3940(87)90628-8 2442675

[B62] Hájos N. Papp E. C. S. Acsády L. Levey A. I. Freund T. F. (1998). Distinct interneuron types express m2 muscarinic receptor immunoreactivity on their dendrites or axon terminals in the hippocampus. Neuroscience 82, 355–376. 10.1016/S0306-4522(97)00300-X 9466448

[B63] Hamilton T. J. Wheatley B. M. Sinclair D. B. Bachmann M. Larkum M. E. Colmers W. F. (2010). Dopamine modulates synaptic plasticity in dendrites of rat and human dentate granule cells. Proc. Natl. Acad. Sci. U.S.A. 107, 18185–18190. 10.1073/pnas.1011558107 20921404 PMC2964233

[B64] Hansen N. Manahan-Vaughan D. (2015a). Hippocampal long-term potentiation that is elicited by perforant path stimulation or that occurs in conjunction with spatial learning is tightly controlled by beta-adrenoreceptors and the locus coeruleus. Hippocampus 25, 1285–1298. 10.1002/hipo.22436 25727388 PMC6680149

[B65] Hansen N. Manahan-Vaughan D. (2015b). Locus coeruleus stimulation facilitates long-term depression in the dentate gyrus that requires activation of β-adrenergic receptors. Cereb. Cortex 25, 1889–1896. 10.1093/cercor/bht429 24464942 PMC4459289

[B66] Hasselmo M. E. (2006). The role of acetylcholine in learning and memory. Curr. Opin. Neurobiol. 16, 710–715. 10.1016/j.conb.2006.09.002 17011181 PMC2659740

[B67] Hasselmo M. E. Schnell E. Barkai E. (1995). Dynamics of learning and recall at excitatory recurrent synapses and cholinergic modulation in rat hippocampal region CA3. J. Neurosci. 15, 5249–5262. 7623149 10.1523/JNEUROSCI.15-07-05249.1995 PMC6577857

[B68] Hebb D. O. (1949). The Organization of Behavior. New York, NY: John Wiley & Sons, Inc.

[B69] Hofmann M. E. Frazier C. J. (2010). Muscarinic receptor activation modulates the excitability of hilar mossy cells through the induction of an afterdepolarization. Brain Res. 1318, 42–51. 10.1016/j.brainres.2010.01.011 20079344 PMC2850114

[B70] Hofmann M. E. Nahir B. Frazier C. J. (2006). Endocannabinoid-mediated depolarization-induced suppression of inhibition in hilar mossy cells of the rat dentate gyrus. J. Neurophysiol. 96, 2501–2512. 10.1152/jn.00310.2006 16807350

[B71] Hofmann M. E. Nahir B. Frazier C. J. (2008). Excitatory afferents to CA3 pyramidal cells display differential sensitivity to CB1 dependent inhibition of synaptic transmission. Neuropharmacology 55, 1140–1146. 10.1016/j.neuropharm.2008.07.007 18675282 PMC2610849

[B72] Hopkins W. F. Johnston D. (1984). Frequency-dependent noradrenergic modulation of long-term potentiation in the hippocampus. Science 226, 350–352. 10.1126/science.6091272 6091272

[B73] Hopkins W. F. Johnston D. (1988). Noradrenergic enhancement of long term potentiation at mossy fiber synapses in the hippocampus. J. Neurophysiol. 59, 667–687. 2832552 10.1152/jn.1988.59.2.667

[B74] Hörtnagl H. Berger M. L. Sperk G. Pifl C. (1991). Regional heterogeneity in the distribution of neurotransmitter markers in the rat hippocampus. Neuroscience 45, 261–272. 10.1016/0306-4522(91)90224-C 1684835

[B75] Hu H. Real E. Takamiya K. Kang M.-G. Ledoux J. Huganir R. L.. (2007). Emotion enhances learning via norepinephrine regulation of AMPA-receptor trafficking. Cell 131, 160–173. 10.1016/j.cell.2007.09.017 17923095

[B76] Huang Y.-Y. Kandel E. R. (1996). Modulation of both the early and the late phase of mossy fiber LTP by the activation of β-adrenergic receptors. Neuron 16, 611–617. 10.1016/S0896-6273(00)80080-X 8785058

[B77] Hummos A. Franklin C. C. Nair S. S. (2014). Intrinsic mechanisms stabilize encoding and retrieval circuits differentially in a hippocampal network model. Hippocampus 19, 1–19. 10.1002/hipo.22324 PMC9121438 24978936

[B78] Hunsaker M. R. Rogers J. L. Kesner R. P. (2007). Behavioral characterization of a transection of dorsal CA3 subcortical efferents: comparison with scopolamine and physostigmine infusions into dorsal CA3. Neurobiol. Learn. Mem. 88, 127–136. 10.1016/j.nlm.2007.01.006 17350296 PMC2095787

[B79] Hunsaker M. R. Rosenberg J. S. Kesner R. P. (2008). The role of the dentate gyrus, CA3a,b, and CA3c for detecting spatial and environmental novelty. Hippocampus 18, 1064–1073. 10.1002/hipo.20464 18651615

[B80] Jacobs B. L. Fornal C. A. (1999). Activity of serotonergic neurons in behaving animals. Neuropsychopharmacology 21(2 Suppl.), 9S–15S. 10.1016/S0893-133X(99)00012-3 10432483

[B81] Jerman T. Kesner R. P. Hunsaker M. R. (2006). Disconnection analysis of CA3 and DG in mediating encoding but not retrieval in a spatial maze learning task. Learn. Mem. 13, 458–464. 10.1101/lm.246906 16882862 PMC1538923

[B82] Jochems A. Yoshida M. (2013). Persistent firing supported by an intrinsic cellular mechanism in hippocampal CA3 pyramidal cells. Eur. J. Neurosci. 38, 2250–2259. 10.1111/ejn.12236 23651161

[B83] Jochems A. Yoshida M. (2015). A robust *in vivo*-like persistent firing supported by a hybrid of intracellular and synaptic mechanisms. PLoS ONE 10, 1–22. 10.1371/journal.pone.0123799 25901969 PMC4406621

[B84] John D. Shelukhina I. Yanagawa Y. Deuchars J. Henderson Z. (2015). Functional α7 nicotinic receptors are expressed on immature granule cells of the postnatal dentate gyrus. Brain Res. 1601, 15–30. 10.1016/j.brainres.2014.12.041 25553616 PMC4350854

[B85] Jones S. Yakel J. L. (1997). Functional nicotinic ACh receptors on interneurones in the rat hippocampus. J. Physiol. 504, 603–610. 10.1111/j.1469-7793.1997.603bd.x 9401968 PMC1159964

[B86] Jung M. W. McNaughton B. L. (1993). Spatial selectivity of unit activity in the hippocampal granular layer. Hippocampus 3, 165–182. 10.1002/hipo.450030209 8353604

[B87] Jurgens C. W. D. Rau K. E. Knudson C. A. King J. D. Carr P. A. Porter J. E.. (2005). β1 adrenergic receptor-mediated enhancement of hippocampal CA3 network activity. J. Pharmacol. Exp. Ther. 314, 552–560. 10.1124/jpet.105.085332 15908512

[B88] Karunakaran S. Chowdhury A. Donato F. Quairiaux C. Michel C. M. Caroni P. (2016). PV plasticity sustained through D1/5 dopamine signaling required for long-term memory consolidation. Nat. Neurosci. 19, 454–464. 10.1038/nn.4231 26807952

[B89] Katsuki H. Izumi Y. Zorumski C. F. (1997). Noradrenergic regulation of synaptic plasticity in the hippocampal CA1 region. J. Neurophysiol. 77, 3013–3020. 9212253 10.1152/jn.1997.77.6.3013

[B90] Kheirbek M. A. Drew L. J. Burghardt N. S. Costantini D. O. Tannenholz L. Ahmari S. E.. (2013). Differential control of learning and anxiety along the dorsoventral axis of the dentate gyrus. Neuron 77, 955–968. 10.1016/j.neuron.2012.12.038 23473324 PMC3595120

[B91] Kitt C. A. Höhmann C. Coyle J. T. Price D. L. (1994). Cholinergic innervation of mouse forebrain structures. J. Comp. Neurol. 341, 117–129. 10.1002/cne.903410110 8006218

[B92] Kobayashi K. Ikeda Y. Haneda E. Suzuki H. (2008). Chronic fluoxetine bidirectionally modulates potentiating effects of serotonin on the hippocampal mossy fiber synaptic transmission. J. Neurosci. 28, 6272–6280. 10.1523/JNEUROSCI.1656-08.2008 18550770 PMC6670533

[B93] Kobayashi K. Ikeda Y. Suzuki H. (2006). Locomotor activity correlates with modifications of hippocampal mossy fibre synaptic transmission. Eur. J. Neurosci. 24, 1867–1873. 10.1111/j.1460-9568.2006.05079.x 17040477

[B94] Kobayashi K. Poo M. M. (2004). Spike train timing-dependent associative modification of hippocampal CA3 recurrent synapses by mossy fibers. Neuron 41, 445–454. 10.1016/S0896-6273(03)00873-0 14766182

[B95] Kobayashi K. Suzuki H. (2007). Dopamine selectively potentiates hippocampal mossy fiber to CA3 synaptic transmission. Neuropharmacology 52, 552–561. 10.1016/j.neuropharm.2006.08.026 17049952

[B96] Kulla A. Manahan-Vaughan D. (2000). Depotentiation in the dentate gyrus of freely moving rats is modulated by D1/D5 dopamine receptors. Cereb. Cortex 10, 614–620. 10.1093/cercor/10.6.614 10859139

[B97] Kunec S. Hasselmo M. E. Kopell N. J. (2005). Encoding and retrieval in the CA3 region of the hippocampus: a model of theta-phase separation. J. Neurophysiol. 94, 70–82. 10.1152/jn.00731.2004 15728768

[B98] Kwon O. B. Paredes D. Gonzalez C. M. Neddens J. Hernandez L. Vullhorst D.. (2008). Neuregulin-1 regulates LTP at CA1 hippocampal synapses through activation of dopamine D4 receptors. Proc. Natl. Acad. Sci. U.S.A. 105, 15587–15592. 10.1073/pnas.0805722105 18832154 PMC2563131

[B99] Lacaille J. C. Schwartzkroin P. A. (1988). Intracellular responses of rat hippocampal granule cells *in vitro* to discrete applications of norepinephrine. Neurosci. Lett. 89, 176–181. 10.1016/0304-3940(88)90377-1 3393295

[B100] Lee I. Kesner R. P. (2004). Encoding versus retrieval of spatial memory: Double dissociation between the dentate gyrus and the perforant path inputs into CA3 in the dorsal hippocampus. Hippocampus 14, 66–76. 10.1002/hipo.10167 15058484

[B101] Lemon N. Manahan-Vaughan D. (2012). Dopamine D1/D5 receptors contribute to *de novo* hippocampal LTD mediated by novel spatial exploration or locus coeruleus activity. Cereb. Cortex 22, 2131–2138. 10.1093/cercor/bhr297 22038910 PMC3412443

[B102] Lengyel M. Dayan P. (2007). Uncertainty, phase and oscillatory hippocampal recall, in Advances in Neural Information Processing Systems 19, eds Schölkopf B. Platt J. C. Hoffman T. (MIT Press), 833–840. Available online at: http://papers.nips.cc/paper/3102-uncertainty-phase-and-oscillatory-hippocampal-recall

[B103] Lengyel M. Kwag J. Paulsen O. Dayan P. (2005). Matching storage and recall: hippocampal spike timing-dependent plasticity and phase response curves. Nat. Neurosci. 8, 1677–1683. 10.1038/nn1561 16261136

[B104] Leranth C. Hajszan T. (2007). Extrinsic afferent systems to the dentate gyrus. Prog. Brain Res. 163, 63–84. 10.1016/S0079-6123(07)63004-0 17765712 PMC1989689

[B105] Leutgeb J. K. Leutgeb S. Moser M.-B. Moser E. I. (2007). Pattern separation in the dentate gyrus and CA3 of the hippocampus. Science 315, 961–966. 10.1126/science.1135801 17303747

[B106] Levey A. I. Edmunds S. M. Koliatsos V. Wiley R. G. Heilman C. J. (1995). Expression of m1-m4 muscarinic acetylcholine receptor proteins in rat hippocampus and regulation by cholinergic innervation. J. Neurosci. 15(5 Pt 2), 4077–4092. 7751967 10.1523/JNEUROSCI.15-05-04077.1995 PMC6578239

[B107] Li Q. H. Nakadate K. Tanaka-Nakadate S. Nakatsuka D. Cui Y. Watanabe Y. (2004). Unique expression patterns of 5-HT2A and 5-HT2C receptors in the rat brain during postnatal development: western blot and immunohistochemical analyses. J. Comp. Neurol. 469, 128–140. 10.1002/cne.11004 14689478

[B108] Liotta A. Caliskan G. ul Haq R. Hollnagel J. O. Rosler A. Heinemann U.. (2011). Partial disinhibition is required for transition of stimulus-induced sharp wave-ripple complexes into recurrent epileptiform discharges in rat hippocampal slices. J. Neurophysiol. 105, 172–187. 10.1152/jn.00186.2010 20881199

[B109] Lisman J. E. (1999). Relating hippocampal circuitry to function: recall of memory sequences by reciprocal dentate-CA3 interactions. Neuron 22, 233–242. 10.1016/S0896-6273(00)81085-5 10069330

[B110] Lisman J. E. Otmakhova N. A. (2001). Storage, recall, and novelty detection of sequences by the hippocampus: elaborating on the SOCRATIC model to account for normal and aberrant effects of dopamine. Hippocampus 11, 551–568. 10.1002/hipo.1071 11732708

[B111] Losonczy A. Biró A. A. Nusser Z. (2004). Persistently active cannabinoid receptors mute a subpopulation of hippocampal interneurons. Proc. Natl. Acad. Sci. U.S.A. 101, 1362–1367. 10.1073/pnas.0304752101 14734812 PMC337058

[B112] Luo A. H. Tahsili-Fahadan P. Wise R. A. Lupica C. R. Aston-Jones G. (2011). Linking context with reward: a functional circuit from hippocampal CA3 to ventral tegmental area. Science 333, 353–357. 10.1126/science.1204622 21764750 PMC3150711

[B113] Lüscher C. Jan L. Y. Stoffel M. Malenka R. C. Nicoll R. A. (1997). G protein-coupled inwardly rectifying K+ channels (GIRKs) mediate postsynaptic but not presynaptic transmitter actions in hippocampal neurons. Neuron 19, 687–695. 10.1016/S0896-6273(00)80381-5 9331358

[B114] Lynch M. A. Bliss T. V. P. (1986). Noradrenaline modulates the release of [14C]glutamate from dentate but not from CA1/CA3 slices of rat hippocampus. Neuropharmacology 25, 493–498. 10.1016/0028-3908(86)90173-5 2874519

[B115] Maccaferri G. Tóth K. McBain C. J. (1998). Target-specific expression of presynaptic mossy fiber plasticity. Science 279, 1368–1370. 10.1126/science.279.5355.1368 9478900

[B116] Madison D. V. Nicoll R. A. (1982). Noradrenaline blocks accommodation of pyramidal cell discharge in the hippocampus. Nature 299, 636–638. 10.1038/299636a0 6289127

[B117] Madison D. V. Nicoll R. A. (1986). Actions of noradrenaline recorded intracellularly in rat hippocampal CA1 pyramidal neurons, *in vitro*. J. Physiol. 372, 221–244. 10.1113/jphysiol.1986.sp016006 2873241 PMC1192760

[B118] Maeda T. Kaneko S. Satoh M. (1994). Inhibitory influence via 5-HT3 receptors on the induction of LTP in mossy fiber-CA3 system of guinea-pig hippocampal slices. Neurosci. Res. 18, 277–282. 10.1016/0168-0102(94)90163-5 8190370

[B119] Manuel-Apolinar L. Rocha L. Pascoe D. Castillo E. Castillo C. Meneses A. (2005). Modifications of 5-HT4 receptor expression in rat brain during memory consolidation. Brain Res. 1042, 73–81. 10.1016/j.brainres.2005.02.020 15823255

[B120] Marín-Burgin A. Mongiat L. Pardi M. Schinder A. F. (2012). Unique processing during a period of high excitation/inhibition balance in adult-born neurons. Science 335, 1238–1242. 10.1126/science.1214956 22282476 PMC3385415

[B121] Marr D. (1971). Simple memory: a theory for archicortex. Philos. Trans. R. Soc. Lond. B Biol. Sci. 262, 23–81. 10.1098/rstb.1971.0078 4399412

[B122] Martinello K. Huang Z. Lujan R. Tran B. Watanabe M. Cooper E. C.. (2015). Cholinergic afferent stimulation induces axonal function plasticity in adult hippocampal granule cells. Neuron 85, 346–363. 10.1016/j.neuron.2014.12.030 25578363 PMC4306544

[B123] McHugh T. J. Jones M. W. Quinn J. J. Balthasar N. Coppari R. Elmquist J. K.. (2007). Dentate gyrus NMDA receptors mediate rapid pattern separation in the hippocampal network. Science 317, 94–99. 10.1126/science.1140263 17556551

[B124] McIntyre C. K. Hatfield T. McGaugh J. L. (2002). Amygdala norepinephrine levels after training predict inhibitory avoidance retention performance in rats. Eur. J. Neurosci. 16, 1223–1226. 10.1046/j.1460-9568.2002.02188.x 12405982

[B125] McMahon L. L. Kauer J. A. (1997). Hippocampal interneurons are excited via serotonin-gated ion channels. J. Neurophysiol. 78, 2493–2502. 9356400 10.1152/jn.1997.78.5.2493

[B126] McNaughton B. L. Morris R. G. M. (1987). Hippocampal synaptic enhancement and information storage within a distributed memory system. Trends Neurosci. 10, 408–415. 10.1016/0166-2236(87)90011-7

[B127] McQuiston A. R. Madison D. V. (1999). Nicotinic receptor activation excites distinct subtypes of interneurons in the rat hippocampus. J. Neurosci. 19, 2887–2896. 10191306 10.1523/JNEUROSCI.19-08-02887.1999 PMC6782295

[B128] Meeter M. Murre J. M. J. Talamini L. M. (2004). Mode shifting between storage and recall based on novelty detection in oscillating hippocampal circuits. Hippocampus 14, 722–741. 10.1002/hipo.10214 15318331

[B129] Meeter M. Talamini L. Schmitt J. A. Riedel W. J. (2006). Effects of 5-HT on memory and the hippocampus: model and data. Neuropsychopharmacology 31, 712–720. 10.1038/sj.npp.1300869 16132065

[B130] Millan M. J. Marin P. Bockaert J. Mannoury la Cour C. (2008). Signaling at G-protein-coupled serotonin receptors: recent advances and future research directions. Trends Pharmacol. Sci. 29, 454–464. 10.1016/j.tips.2008.06.007 18676031

[B131] Milner T. A. Bacon C. E. (1989). GABAergic neurons in the rat hippocampal formation: ultrastructure and synaptic relationships with catecholaminergic terminals. J. Neurosci. 9, 3410–3427. 2795131 10.1523/JNEUROSCI.09-10-03410.1989 PMC6569896

[B132] Milner T. A. Shah P. Pierce J. P. (2000). β-Adrenergic receptors primarily are located on the dendrites of granule cells and interneurons but also are found on astrocytes and a few presynaptic profiles in the rat dentate gyrus. Synapse 36, 178–193. 10.1002/(SICI)1098-2396(20000601)36:3<178::AID-SYN3>3.0.CO;2-6 10819898

[B133] Mishra R. K. Kim S. Guzman S. J. Jonas P. (2016). Symmetric spike timing-dependent plasticity at CA3-CA3 synapses optimizes storage and recall in autoassociative networks. Nat. Commun. 7:11552. 10.1038/ncomms11552 27174042 PMC4869174

[B134] Missale C. Nash S. R. Robinson S. W. Jaber M. Caron M. G. (1998). Dopamine receptors: from structure to function. Physiol. Rev. 78, 189–225. 9457173 10.1152/physrev.1998.78.1.189

[B135] Mistry R. Dennis S. Frerking M. Mellor J. R. (2011). Dentate gyrus granule cell firing patterns can induce mossy fiber long-term potentiation *in vitro*. Hippocampus 21, 1157–1168. 10.1002/hipo.20815 20635414 PMC3081527

[B136] Miyawaki T. Norimoto H. Ishikawa T. Watanabe Y. Matsuki N. Ikegaya Y. (2014). Dopamine receptor activation reorganizes neuronal ensembles during hippocampal sharp waves *in vitro*. PLoS ONE 9:e104438. 10.1371/journal.pone.0104438 25089705 PMC4121245

[B137] Mlinar B. Stocca G. Corradetti R. (2015). Endogenous serotonin facilitates hippocampal long-term potentiation at CA3/CA1 synapses. J. Neural Transm. 122, 177–185. 10.1007/s00702-014-1246-7 24872079

[B138] Mohler E. G. Shacham S. Noiman S. Lezoualc'h F. Robert S. Gastineau M.. (2007). VRX-03011, a novel 5-HT4 agonist, enhances memory and hippocampal acetylcholine efflux. Neuropharmacology 53, 563–573. 10.1016/j.neuropharm.2007.06.016 17692343

[B139] Mori M. Abegg M. H. Gähwiler B. H. Gerber U. (2004). A frequency-dependent switch from inhibition to excitation in a hippocampal unitary circuit. Nature 431, 453–456. 10.1038/nature02854 15386013

[B140] Mori M. Gähwiler B. H. Gerber U. (2007). Recruitment of an inhibitory hippocampal network after bursting in a single granule cell. Proc. Natl. Acad. Sci. U.S.A. 104, 7640–7645. 10.1073/pnas.0702164104 17438288 PMC1863441

[B141] Müller W. Connor J. A. (1991). Dendritic spines as individual neuronal compartments for synaptic Ca2+ responses. Nature 354, 73–76. 10.1038/354073a0 1682815

[B142] Nahir B. Bhatia C. Frazier C. J. (2007). Presynaptic inhibition of excitatory afferents to hilar mossy cells. J. Neurophysiol. 97, 4036–4047. 10.1152/jn.00069.2007 17442771

[B143] Nakashiba T. (2008). Transgenic inhibition of synaptic transmission reveals role of CA3 output in hippocampal learning. Science 319, 1260–1264. 10.1126/science.1151120 18218862

[B144] Nakashiba T. Cushman J. D. Pelkey K. A. Renaudineau S. Buhl D. L. McHugh T. J.. (2012). Young dentate granule cells mediate pattern separation, whereas old granule cells facilitate pattern completion. Cell 149, 188–201. 10.1016/j.cell.2012.01.046 22365813 PMC3319279

[B145] Nakazawa K. McHugh T. J. Wilson M. A. Tonegawa S. (2004). NMDA receptors, place cells and hippocampal spatial memory. Nat. Rev. Neurosci. 5, 361–372. 10.1038/nrn1385 15100719

[B146] Nissen W. Szabo A. Somogyi J. Somogyi P. Lamsa K. P. (2010). Cell type-specific long-term plasticity at glutamatergic synapses onto hippocampal interneurons expressing either parvalbumin or CB1 cannabinoid receptor. J. Neurosci. 30, 1337–1347. 10.1523/JNEUROSCI.3481-09.2010 20107060 PMC2817897

[B147] Nolan C. R. Wyeth G. Milford M. Wiles J. (2011). The race to learn: spike timing and STDP can coordinate learning and recall in CA3. Hippocampus 21, 647–660. 10.1002/hipo.20777 20232384

[B148] Nozaki K. Kubo R. Furukawa Y. (2016). Serotonin modulates the excitatory synaptic transmission in the dentate granule cells. J. Neurophysiol. 115, 2997–3007. 10.1152/jn.00064.2016 26961099 PMC4946589

[B149] Okuhara D. Y. Beck S. G. (1994). 5-HT1A receptor linked to inward-rectifying potassium current in hippocampal CA3 pyramidal cells. J. Neurophysiol. 71, 2161–2167. 7931509 10.1152/jn.1994.71.6.2161

[B150] Ondrejcak T. Wang Q. Kew J. N. C. Virley D. J. Upton N. Anwyl R.. (2012). Activation of α7 nicotinic acetylcholine receptors persistently enhances hippocampal synaptic transmission and prevents A α7-mediated inhibition of LTP in the rat hippocampus. Eur. J. Pharmacol. 677, 63–70. 10.1016/j.ejphar.2011.12.008 22200627

[B151] O'Reilly R. C. McClelland J. L. (1994). Hippocampal conjunctive encoding, storage, and recall: avoiding a trade-off. Hippocampus 4, 661–682. 7704110 10.1002/hipo.450040605

[B152] Parfitt K. D. Doze V. A. Madison D. V. Browning M. D. (1992). Isoproterenol increases the phosphorylation of the synapsins and increases synaptic transmission in dentate gyrus, but not in area CA1, of the hippocampus. Hippocampus 2, 59–64. 10.1002/hipo.450020108 1339193

[B153] Parfitt K. D. Hoffer B. J. Browing M. D. (1991). Norepinephrine and isoproterenol increase the phosphorylation of synapsin I and synapsin II in dentate slices of young but not aged Fisher 344 rats. Proc. Natl. Acad. Sci. U.S.A. 88, 2361–2365. 10.1073/pnas.88.6.2361 1900942 PMC51231

[B154] Pazos A. Cortés R. Palacios J. M. (1985). Quantitative autoradiographic mapping of serotonin receptors in the rat brain. II. Serotonin-2 receptors. Brain Res. 346, 231–249. 4052777 10.1016/0006-8993(85)90857-1

[B155] Pedarzani P. Storm J. F. (1993). PKA mediates the effects of monoamine transmitters on the K+ current underlying the slow spike frequency adaptation in hippocampal neurons. Neuron 11, 1023–1035. 10.1016/0896-6273(93)90216-E 8274274

[B156] Pelkey K. A. Lavezzari G. Racca C. Roche K. W. McBain C. J. (2005). mGluR7 is a metaplastic switch controlling bidirectional plasticity of feedforward inhibition. Neuron 46, 89–102. 10.1016/j.neuron.2005.02.011 15820696

[B157] Piguet P. Galvan M. (1994). Transient and long-lasting actions of 5-HT on rat dentate gyrus neurones *in vitro*. J. Physiol. 481(Pt 3):629–639. 7707231 10.1113/jphysiol.1994.sp020469 PMC1155906

[B158] Puighermanal E. Biever A. Espallergues J. Gangarossa G. De Bundel D. Valjent E. (2015). Drd2-cre: ribotag mouse line unravels the possible diversity of dopamine d2 receptor-expressing cells of the dorsal mouse hippocampus. Hippocampus 25, 858–875. 10.1002/hipo.22408 25545461

[B159] Radcliffe K. A. Fisher J. L. Gray R. Dani J. A. (1999). Nicotinic modulation of glutamate and GABA synaptic transmission in hippocampal neurons. Ann. N.Y. Acad. Sci. 868, 591–610. 10.1111/j.1749-6632.1999.tb11332.x 10414340

[B160] Ramos B. P. Arnsten A. F. T. (2007). Adrenergic pharmacology and cognition: focus on the prefrontal cortex. Pharmacol. Ther. 113, 523–536. 10.1016/j.pharmthera.2006.11.006 17303246 PMC2151919

[B161] Restivo L. Niibori Y. Mercaldo V. Josselyn S. A. Frankland P. W. (2015). Development of adult-generated cell connectivity with excitatory and inhibitory cell populations in the hippocampus. J. Neurosci. 35, 10600–10612. 10.1523/JNEUROSCI.3238-14.2015 26203153 PMC6605118

[B162] Romo-Parra H. Aceves J. Gutiérrez R. (2005). Tonic modulation of inhibition by dopamine D4 receptors in the rat hippocampus. Hippocampus 15, 254–259. 10.1002/hipo.20049 15476261

[B163] Rosen Z. B. Cheung S. Siegelbaum S. A. (2015). Midbrain dopamine neurons bidirectionally regulate CA3-CA1 synaptic drive. Nat. Neurosci. 18, 1–11. 10.1038/nn.4152 26523642 PMC11186581

[B164] Rüdiger T. Bolz J. (2008). Acetylcholine influences growth cone motility and morphology of developing thalamic axons. Cell Adhes. Migr. 2, 30–37. 10.4161/cam.2.1.5909 19262162 PMC2635000

[B165] Ruediger S. Vittori C. Bednarek E. Genoud C. Strata P. Sacchetti B.. (2011). Learning-related feedforward inhibitory connectivity growth required for memory precision. Nature 473, 514–518. 10.1038/nature09946 21532590

[B166] Ruiz A. Campanac E. Scott R. S. Rusakov D. a. Kullmann D. M. (2010). Presynaptic GABAA receptors enhance transmission and LTP induction at hippocampal mossy fiber synapses. Nat. Neurosci. 13, 431–438. 10.1038/nn.2512 20305647 PMC2898498

[B167] Salin P. A. Scanziani M. Malenka R. C. Nicoll R. A. (1996). Distinct short-term plasticity at two excitatory synapses in the hippocampus. Proc. Natl. Acad. Sci. U.S.A. 93, 13304–13309. 10.1073/pnas.93.23.13304 8917586 PMC24088

[B168] Sara S. J. (2009). The locus coeruleus and noradrenergic modulation of cognition. Nat. Rev. Neurosci. 10, 211–223. 10.1038/nrn2573 19190638

[B169] Saravanan V. Arabali D. Jochems A. Cui A. X. Gootjes-Dreesbach L. Cutsuridis V.. (2015). Transition between encoding and consolidation/replay dynamics via cholinergic modulation of CAN current: a modeling study. Hippocampus 25, 1052–1070. 10.1002/hipo.22429 25678405

[B170] Sariñana J. Kitamura T. Künzler P. Sultzman L. Tonegawa S. (2014). Differential roles of the dopamine 1-class receptors, D1R and D5R, in hippocampal dependent memory. Proc. Natl. Acad. Sci. U.S.A. 111, 8245–8250. 10.1073/pnas.1407395111 24843151 PMC4050601

[B171] Savin C. Dayan P. Lengyel M. (2014). Optimal recall from bounded metaplastic synapses: predicting functional adaptations in hippocampal area CA3. PLoS Comput. Biol. 10:e1003489. 10.1371/journal.pcbi.1003489 24586137 PMC3937414

[B172] Scanziani M. Gahwiler B. H. Thompson S. M. (1993). Presynaptic inhibition of excitatory synaptic transmission mediated by alpha adrenergic receptors in area CA3 of the rat hippocampus *in vitro*. J. Neurosci. 13, 5393–5401. 7504723 10.1523/JNEUROSCI.13-12-05393.1993 PMC6576403

[B173] Scanziani M. Gahwiler B. H. Thompson S. M. (1995). Presynaptic inhibition of excitatory synaptic transmission by muscarinic and metabotropic glutamate receptor activation in the hippocampus: are Ca2+ channels involved? Neuropharmacology 34, 1549–1557. 8606802 10.1016/0028-3908(95)00119-q

[B174] Scoville W. B. Milner B. (1957). Loss of recent memory after bilateral hippocampal lesions. 1957. J. Neurol. Neurosurg. Psychiatry 20, 11–21. 10.1136/jnnp.20.1.11 13406589 PMC497229

[B175] Segal M. Markram H. Richter-Levin G. (1991). Actions of norepinephrine in the rat hippocampus. Prog. Brain Res. 88, 323–330. 10.1016/S0079-6123(08)63819-4 1667547

[B176] Sharma G. Grybko M. Vijayaraghavan S. (2008). Action potential-independent and nicotinic receptor-mediated concerted release of multiple quanta at hippocampal CA3-mossy fiber synapses. J. Neurosci. 28, 2563–2575. 10.1523/JNEUROSCI.5407-07.2008 18322100 PMC2696816

[B177] Sharma G. Vijayaraghavan S. (2003). Modulation of presynaptic store calcium induces release of glutamate and postsynaptic firing. Neuron 38, 929–939. 10.1016/S0896-6273(03)00322-2 12818178

[B178] Smith C. C. Greene R. W. (2012). CNS dopamine transmission mediated by noradrenergic innervation. J. Neurosci. 32, 6072–6080. 10.1523/JNEUROSCI.6486-11.2012 22553014 PMC3371362

[B179] Sodickson D. L. Bean B. P. (1996). GABAB receptor-activated inwardly rectifying potassium current in dissociated hippocampal CA3 neurons. J. Neurosci. 16, 6374–6385. 8815916 10.1523/JNEUROSCI.16-20-06374.1996 PMC6578909

[B180] Son J.-H. Winzer-Serhan U. H. (2008). Expression of neuronal nicotinic acetylcholine receptor subunit mRNAs in rat hippocampal GABAergic interneurons. J. Comp. Neurol. 511, 286–299. 10.1002/cne.21828 18792073 PMC3271947

[B181] Squire L. R. (1992). Memory and the hippocampus: a synthesis from findings with rats, monkeys, and humans. Psychol. Rev. 99, 195–231. 10.1037/0033-295X.99.2.195 1594723

[B182] Straube T. Frey J. U. (2003). Involvement of β-adrenergic receptors in protein synthesis-dependent late long-term potentiation (LTP) in the dentate gyrus of freely moving rats: the critical role of the LTP induction strength. Neuroscience 119, 473–479. 10.1016/S0306-4522(03)00151-9 12770561

[B183] Suwa B. Bock N. Preusse S. Rothenberger A. Manzke T. (2014). Distribution of serotonin 4(a) receptors in the juvenile rat brain and spinal cord. J. Chem. Neuroanat. 55, 67–77. 10.1016/j.jchemneu.2013.12.004 24412663

[B184] Szabadics J. Soltesz I. (2009). Functional specificity of mossy fiber innervation of GABAergic cells in the hippocampus. J. Neurosci. 29, 4239–4251. 10.1523/JNEUROSCI.5390-08.2009 19339618 PMC6665380

[B185] Szabó G. G. Holderith N. Gulyás A. I. Freund T. F. Hájos N. (2010). Distinct synaptic properties of perisomatic inhibitory cell types and their different modulation by cholinergic receptor activation in the CA3 region of the mouse hippocampus. Eur. J. Neurosci. 31, 2234–2246. 10.1111/j.1460-9568.2010.07292.x 20529124 PMC2916217

[B186] Takeuchi T. Duszkiewicz A. J. Sonneborn A. Spooner P. A. Yamasaki M. Watanabe M.. (2016). Locus coeruleus and dopaminergic consolidation of everyday memory. Nature 537, 357–362. 10.1038/nature19325 27602521 PMC5161591

[B187] Tang A.-H. Karson M. A. Nagode D. A. McIntosh J. M. Uebele V. N. Renger J. J.. (2011). Nerve terminal nicotinic acetylcholine receptors initiate quantal GABA release from perisomatic interneurons by activating axonal T-type (Cav3) Ca2+ channels and Ca2+ release from stores. J. Neurosci. 31, 13546–13561. 10.1523/JNEUROSCI.2781-11.2011 21940446 PMC3353409

[B188] Tigaret C. M. Olivo V. Sadowski J. H. Ashby M. C. Mellor J. R. (2016). Coordinated activation of distinct Ca2+ sources and metabotropic glutamate receptors encodes Hebbian synaptic plasticity. Nat. Commun. 7, 1–15. 10.1038/ncomms10289 PMC4735496 26758963

[B189] Torborg C. L. Nakashiba T. Tonegawa S. McBain C. J. (2010). Control of CA3 output by feedforward inhibition despite developmental changes in the excitation-inhibition balance. J. Neurosci. 30, 15628–15637. 10.1523/JNEUROSCI.3099-10.2010 21084618 PMC3023412

[B190] Törk I. (1990). Anatomy of the serotonergic system. Ann. N.Y. Acad. Sci. 600, 5–9. 10.1111/j.1749-6632.1990.tb16870.x 2252340

[B191] Toth K. Suares G. Lawrence J. J. Philips-Tansey E. McBain C. J. (2000). Differential mechanisms of transmission at three types of mossy fiber synapse. J. Neurosci. 20, 8279–8289. Available online at: http://www.jneurosci.org/content/20/22/8279 11069934 10.1523/JNEUROSCI.20-22-08279.2000 PMC6773175

[B192] Treves A. Rolls E. T. (1994). Computational analysis of the role of the hippocampus in memory. Hippocampus 4, 374–391. 10.1002/hipo.450040319 7842058

[B193] Treviño M. Vivar C. Gutiérrez R. (2011). Excitation-inhibition balance in the CA3 network–neuronal specificity and activity-dependent plasticity. Eur. J. Neurosci. 33, 1771–1785. 10.1111/j.1460-9568.2011.07670.x 21501253

[B194] Twarkowski H. Hagena H. Manahan-Vaughan D. (2016). The 5-hydroxytryptamine4 receptor enables differentiation of informational content and encoding in the hippocampus. Hippocampus 26, 875–891. 10.1002/hipo.22569 26800645 PMC5067691

[B195] Tzounopoulos T. Janz R. Südhof T. C. Nicoll R. A. Malenka R. C. (1998). A role for cAMP in long-term depression at hippocampal mossy fiber synapses. Neuron 21, 837–845. 10.1016/S0896-6273(00)80599-1 9808469

[B196] Ul Haq R. Anderson M. L. Hollnagel J. O. Worschech F. Sherkheli M. A. Behrens C. J.. (2016). Serotonin dependent masking of hippocampal sharp wave ripples. Neuropharmacology 101, 188–203. 10.1016/j.neuropharm.2015.09.026 26409781

[B197] Ul Haq R. Liotta A. Kovacs R. Rosler A. Jarosch M. J. Heinemann U.. (2012). Adrenergic modulation of sharp wave-ripple activity in rat hippocampal slices. Hippocampus 22, 516–533. 10.1002/hipo.20918 21254303

[B198] Vankov A. Herve-Minvielle A. Sara S. J. (1995). Response to novelty and its rapid habituation in locus coeruleus neurons of the freely exploring rat. Eur. J. Neurosci. 7, 1180–1187. 10.1111/j.1460-9568.1995.tb01108.x 7582091

[B199] Varga V. Losonczy A. Zemelman B. V. Borhegyi Z. Nyiri G. Domonkos A.. (2009). Fast synaptic subcortical control of hippocampal circuits. Science 326, 449–453. 10.1126/science.1178307 19833972

[B200] Vilaró M. T. Cortés R. Gerald C. Branchek T. A. Palacios J. M. Mengod G. (1996). Localization of 5-HT4 receptor mRNA in rat brain by *in situ* hybridization histochemistry. Mol. Brain Res. 43, 356–360. 10.1016/S0169-328X(96)00248-3 9037555

[B201] Vilaró M. T. Cortés R. Mengod G. (2005). Serotonin 5-HT4 receptors and their mRNAs in rat and guinea pig brain: distribution and effects of neurotoxic lesions. J. Comp. Neurol. 484, 418–439. 10.1002/cne.20447 15770652

[B202] Villani F. Johnston D. (1993). Serotonin inhibits induction of long-term potentiation at commissural synapses in hippocampus. Brain Res. 606, 304–308. 10.1016/0006-8993(93)90998-3 8387861

[B203] Vogels T. P. Sprekeler H. Zenke F. Clopath C. Gerstner W. (2011). Inhibitory plasticity balances excitation and inhibition in sensory pathways and memory networks. Science 334, 1569–1573. 10.1126/science.1211095 22075724

[B204] Vogt K. E. Regehr W. G. (2001). Cholinergic modulation of excitatory synaptic transmission in the CA3 area of the hippocampus. J. Neurosci. 21, 75–83. Available online at: http://www.jneurosci.org/content/21/1/75 11150322 10.1523/JNEUROSCI.21-01-00075.2001 PMC6762445

[B205] Walling S. G. Brown R. A. Miyasaka N. Yoshihara Y. Harley C. W. (2012). Selective wheat germ agglutinin (WGA) uptake in the hippocampus from the locus coeruleus of dopamine-β-hydroxylase-WGA transgenic mice. Front. Behav. Neurosci. 6 23. 10.3389/fbeh.2012.00023 22654744 PMC3361128

[B206] Walling S. G. Harley C. W. (2004). Locus ceruleus activation initiates delayed synaptic potentiation of perforant path input to the dentate gyrus in awake rats: a novel β-adrenergic- and protein synthesis-dependent mammalian plasticity mechanism. J. Neurosci. 24, 598–604. 10.1523/JNEUROSCI.4426-03.2004 14736844 PMC6729256

[B207] Wang J. K. T. Andrews H. Thukral V. (1992). Presynaptic glutamate receptors regulate noradrenaline release from isolated nerve terminals. J. Neurochem. 58, 204–211. 10.1111/j.1471-4159.1992.tb09297.x 1345765

[B208] Welsby P. J. Rowan M. Anwyl R. (2006). Nicotinic receptor-mediated enhancement of long-term potentiation involves activation of metabotropic glutamate receptors and ryanodine-sensitive calcium stores in the dentate gyrus. Eur. J. Neurosci. 24, 3109–3118. 10.1111/j.1460-9568.2006.05187.x 17156372

[B209] Welsby P. J. Rowan M. J. Anwyl R. (2009). Intracellular mechanisms underlying the nicotinic enhancement of LTP in the rat dentate gyrus. Eur. J. Neurosci. 29, 65–75. 10.1111/j.1460-9568.2008.06562.x 19077124

[B210] West M. J. Slomianka L. Gundersen H. J. G. (1991). Unbiased stereological estimation of the total number of neurons in the subdivisions of the rat hippocampus using the optical fractionator. Anat. Rec. 231, 482–497. 10.1002/ar.1092310411 1793176

[B211] Wiescholleck V. Manahan-Vaughan D. (2014). Antagonism of D1/D5 receptors prevents long-term depression (LTD) and learning-facilitated LTD at the perforant path-dentate gyrus synapse in freely behaving rats. Hippocampus 24, 1615–1622. 10.1002/hipo.22340 25112177

[B212] Wilke S. A. Antonios J. K. Bushong E. A. Badkoobehi A. Malek E. Hwang M.. (2013). Deconstructing complexity: serial block-face electron microscopic analysis of the hippocampal mossy fiber synapse. J. Neurosci. 33, 507–522. 10.1523/JNEUROSCI.1600-12.2013 23303931 PMC3756657

[B213] Williams S. Johnston D. (1988). Muscarinic depression of long-term potentiation in CA3 hippocampal neurons. Science 242, 84–87. 10.1126/science.2845578 2845578

[B214] Williams S. Johnston D. (1990). Muscarinic depression of synaptic transmission at the hippocampal mossy fiber synapse. J. Neurophysiol. 64, 1089–1097. 2175351 10.1152/jn.1990.64.4.1089

[B215] Yang K. Dani J. A. (2014). Dopamine D1 and D5 receptors modulate spike timing-dependent plasticity at medial perforant path to dentate granule cell synapses. J. Neurosci. 34, 15888–15897. 10.1523/JNEUROSCI.2400-14.2014 25429131 PMC4244463

[B216] Yi F. Ball J. Stoll K. E. Satpute V. C. Mitchell S. M. Pauli J. L.. (2014). Direct excitation of parvalbumin-positive interneurons by M1 muscarinic acetylcholine receptors: roles in cellular excitability, inhibitory transmission and cognition. J. Physiol. 592(Pt 16), 3463–3494. 10.1113/jphysiol.2014.275453 24879872 PMC4229343

[B217] Zalutsky R. A. Nicoll R. A. (1990). Comparison of two forms of long-term potentiation in single hippocampal neurons. Science 248, 1619–1624. 10.1126/science.2114039 2114039

[B218] Zhang G. Stackman R. W. (2015). The role of serotonin 5-HT2A receptors in memory and cognition. Front. Pharmacol. 6 225. 10.3389/fhar.2015.00225 26500553 PMC4594018

